# The RP11-417E7.1/THBS2 signaling pathway promotes colorectal cancer metastasis by activating the Wnt/β-catenin pathway and facilitating exosome-mediated M2 macrophage polarization

**DOI:** 10.1186/s13046-024-03107-7

**Published:** 2024-07-17

**Authors:** Yunze Liu, Heng Lv, Xin Liu, Lei Xu, Tiankang Li, Hui Zhou, Hongmei Zhu, Chuanchuan Hao, Changwei Lin, Yi Zhang

**Affiliations:** 1 Department of Gastrointestinal Surgery, Affiliated Hospital of Xuzhou Medical University, Xuzhou, 221000 China; 2 Institute of Digestive Diseases, Xuzhou Medical University, Xuzhou, 221000 China; 3 Department of Endocrinology, Affiliated Hospital of Xuzhou Medical University, Xuzhou, 221000 China; 4 Department of Biology and Biochemistry, Center for Nuclear Receptors and Cell Signaling, University of Houston, Houston, TX 77204 USA; 5 Department of Gastrointestinal Surgery, The Third XiangYa Hospital of Central South University, Changsha, Hunan 410013 China; 6 Department of Traditional Chinese Medicine, Affiliated Hospital of Xuzhou Medical University, Xuzhou, 221000 China; 7 Department of Epigenetics and Molecular Carcinogenesis, University of Texas MD Anderson Cancer Center, Houston, TX 77030 USA

**Keywords:** RP11-417E7.1, Colorectal cancer, Metastasis, Macrophage polarization, Wnt/β-catenin, Netropsin

## Abstract

**Background:**

Metastasis is the major cause of colorectal cancer (CRC) mortality. Emerging evidence suggests that long noncoding RNAs (lncRNAs) drive cancer metastasis and that their regulatory pathways could be targeted for preventing metastasis. However, the underlying mechanisms of lncRNAs in CRC metastasis remain poorly understood.

**Methods:**

Microarray analysis was used to screen for differentially expressed lncRNAs. Transwell assays, fibronectin cell adhesion assays, and mouse metastasis models were utilized to evaluate the metastatic capacities of CRC in vitro and in vivo. Chromatin isolation by RNA purification, chromatin immunoprecipitation and chromosome conformation capture were applied to investigate the underlying mechanism involved. qRT‒PCR and transmission electron microscopy were performed to confirm macrophage polarization and the presence of cancer-derived exosomes.

**Results:**

The lncRNA RP11-417E7.1 was screened and identified as a novel metastasis-associated lncRNA that was correlated with a poor prognosis. RP11-417E7.1 enhances the metastatic capacity of CRC cells in vivo and in vitro. Mechanistically, RP11-417E7.1 binding with High mobility group A1 (HMGA1) promotes neighboring thrombospondin 2 (THBS2) transcription via chromatin loop formation between its promoter and enhancer, which activates the Wnt/β-catenin signaling pathway and facilitates CRC metastasis. Furthermore, exosomes derived from CRC cells transport THBS2 into macrophages, thereby inducing the M2 polarization of macrophages to sustain the prometastatic microenvironment. Notably, netropsin, a DNA-binding drug, suppresses chromatin loop formation mediated by RP11-417E7.1 at the THBS2 locus and significantly inhibits CRC metastasis in vitro and in vivo.

**Conclusions:**

This study revealed the novel prometastatic function and mechanism of the lncRNA RP11-417E7.1, which provides a potential prognostic indicator and therapeutic target in CRC.

**Supplementary Information:**

The online version contains supplementary material available at 10.1186/s13046-024-03107-7.

## Background

Colorectal cancer (CRC) is the third most common malignancy worldwide [1]. Metastasis is the primary challenge in advanced CRC, and the 5-year survival rate for CRC patients with distant metastasis is less than 15% [2]. However, the specific molecular mechanisms underlying CRC metastasis remain largely unclear, resulting in limited strategies for prevention and treatment [3]. Therefore, a better understanding of the detailed mechanisms for prevention and treatment of CRC metastasis is urgently needed.

The epithelial–mesenchymal transition (EMT) is always the first and key step in the metastasis of different cancers, including CRC [4]. During the EMT, CRC cells lose cell‒cell adhesion and achieve a fibroblast-like morphology, thus increasing their migration and invasion from solid tumors into nearby cell layers [5]. In addition, CRC metastasis can be facilitated by the immunosuppressive tumor microenvironment (TME) [6]. As an important component of the TME, M2-like tumor-associated macrophages (TAMs) increase the migratory and invasive potential of tumor cells by releasing various oncogenic signals, including growth factors, proteases, and cytokines [7, 8].

Long noncoding RNAs (lncRNAs) are genomic transcripts longer than 200 nucleotides that do not encode protein products [9]. Aberrantly expressed lncRNAs have been found to be involved in cancer metastasis [10, 11]. For example, overexpression of the lncRNA LINC00941 can drive CRC metastasis by activating the TGF-β/SMAD2/3 signaling pathway [12]. Overexpression of lncRNA-BX111887 contributes to EMT-dependent lymphatic invasion and distant metastasis in pancreatic cancer by promoting ZEB1 transcription [13]. Understanding dysregulated lncRNAs and their downstream signaling networks is important for revealing the molecular mechanisms of cancer metastasis. However, the crucial lncRNAs involved in CRC metastasis have yet to be fully investigated.

Thrombospondin-2 (THBS2), a member of the calcium-associated glycoprotein family, is widely expressed in various tissues and cell types and plays pivotal roles in essential biological processes such as extracellular matrix formation, cell proliferation, migration, apoptosis, and angiogenesis [14]. However, conflicting findings regarding THBS2 expression and function have been reported across different tumor types [15–17]. In CRC, elevated levels of THBS2 have been associated with reduced survival rates and disease progression. Moreover, THBS2 has been implicated in promoting CRC metastasis [15]; nevertheless, further investigations are required to elucidate the underlying mechanism governing its regulatory role.

In this study, the lncRNA RP11-417E7.1 was significantly overexpressed in CRC tissues with lymph node metastasis or distant metastasis, and high RP11-417E7.1 expression was associated with a poor prognosis. in vivo and in vitro experiments confirmed that RP11-417E7.1 could promote EMT-related metastasis. RP11-417E7.1 enhanced neighboring THBS2 expression by maintaining specific chromatin looping, thus activating the Wnt/β-catenin signaling pathway. We also found that THBS2 could be transferred by exosomes from CRC cells to TAMs to induce the M2 polarization of TAMs, and M2-polarized TAMs promoted CRC cell metastasis via positive feedback. Furthermore, the DNA-binding drug netropsin was shown to inhibit RP11-417E7.1-mediated chromatin loop formation and prevent CRC metastasis.

## Materials and methods

### Cell lines and cell culture

The human normal colon epithelial cell line FHC, CRC cell lines (HCT116, HCT-8, LoVo, DLD1, SW480 and SW620), the human monocyte cell line THP-1, and HEK 293 T cells were obtained from the Cell Bank of the Chinese Academy of Sciences (Shanghai, China). All cell lines were cultured with 10% fetal bovine serum (FBS; Gibco, Waltham, MA, USA) and a 1% antibiotic/antimycotic solution and maintained at 37 °C in an atmosphere of 5% CO_2_. HCT116 cells were grown in McCoy’s 5 A medium (Gibco, Waltham, MA, USA). RPMI-1640 medium (HyClone, Logan, UT, USA) was added to DLD1, LoVo, HT-29, and THP-1 cells. HEK 293-T cells, SW480 cells, and FHC cells were cultured in DMEM (HyClone, Logan, UT, USA).

### Cell transfection

The small hairpin RNA (shRNA) targeting RP11-417E7.1 and negative control (sh-NC), lentiviral RP11-417E7.1 overexpression vector and lentiviral THBS2 overexpression vector were designed and synthesized (GenePharma, Shanghai, China). The shRNAs were delivered by lentiviral infection with lentiviruses following the manufacturer’s instructions. All short interfering RNA (siRNA) sequences targeting THBS2, HMGA1, and YWHAZ and the negative control siRNA (si-NC) were designed and synthesized (GenePharma, Shanghai, China). The siRNAs were transfected into cells with siLentFect Lipid Reagent (Bio-Rad, Hercules, CA, USA), and the cells were harvested for subsequent experiments after 2–3 days. The shRNA and siRNA sequences used are listed in the Supplemental Materials. For overexpression, full-length HMGA1, THBS2, and YWHAZ sequences were cloned and inserted into the plasmid pcDNA3.1, and were synthesized by Genechem (Shanghai, China). For transfection, cells were seeded into 6-well plates and cultured to 80% confluence. Transfection was conducted with Lipofectamine 2000 reagent (Invitrogen, Carlsbad, CA, USA) according to the manufacturer’s protocols. At 48 h posttransfection, the cells were ready for further measurements.

### Statistical analysis

All values are presented as the means ± standard deviations (SDs). The significance of the differences between groups was determined with one-way ANOVA or Student’s t test. Correlations between 2 groups were computed using the χ^2^ test. Spearman’s correlation coefficient was calculated to determine correlations between two groups. The Kaplan–Meier analysis was employed for the survival analysis, and the differences in survival probabilities were estimated using the log-rank test. *p* < 0.05 was considered to indicate statistical significance. Statistical analyses were performed with SPSS version 22.0 (SPSS, Inc., Chicago, IL, USA).

Microarray and RNA-seq assays, qRT‒PCR assays, RNA FISH, IF analysis, RIP assays, chromatin isolation by RNA purification (ChIRP), chromatin immunoprecipitation (ChIP), chromatin conformation capture (3 C) assays, dual-luciferase reporter assays, Western blot analysis and antibodies, coimmunoprecipitation (Co-IP), cell migration and invasion assays, cell adhesion assays, macrophage polarization experiments, exosome isolation and purification, exosome labeling and fluorescence microscopy analyses, immunohistochemistry (IHC), and tumor metastasis experiments in vivo were performed. The details of the above assays are provided in the Supplemental Materials and Methods.

## Results

### High RP11-417E7.1 expression is associated with CRC metastasis and a poor prognosis

**Fig. 1:**
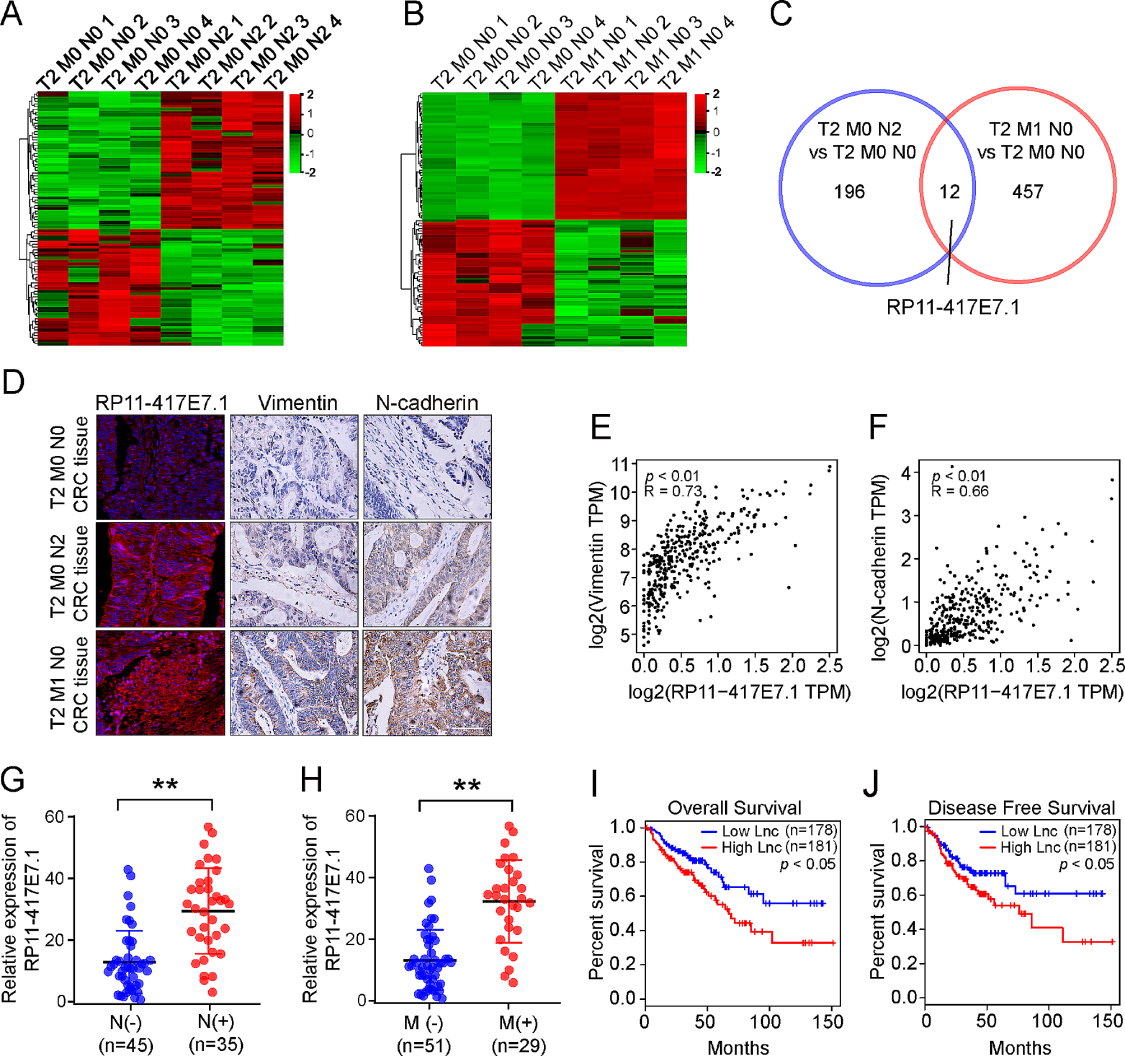
High RP11-417E7.1 expression is associated with CRC metastasis and a poor prognosis. **A**, Heatmap of differentially expressed lncRNAs in T2 CRC tissues without lymph node metastasis or distant metastasis (stage T2M0N0) and in T2 CRC tissues with ≥ 4 lymph node metastases but no distant metastasis (stage T2M0N2). **B**, Heatmap of differentially expressed lncRNAs in T2 CRC tissues without lymph node metastasis or distant metastasis (stage T2M0N0) and tissues from four patients with liver metastasis but no lymph node metastasis (stage T2M1N0). **C**, Venn diagram of the intersection of the above two lists of differentially expressed lncRNAs. **D**, Immunofluorescence staining for RP11-417E7.1 and immunohistochemical staining for vimentin and N-cadherin in T2M0N0, T2M0N2 and T2M1N0 CRC tissues. Scale bar: 50 μm. **E**, Dot plot of the correlation between RP11-417E7.1 and Vimentin mRNA expression in TCGA CRC dataset (from GEPIA2). **F**, Dot plot of the correlation between RP11-417E7.1 and N-cadherin mRNA expression in TCGA CRC dataset (from GEPIA2). **G**, qRT‒PCR analysis of relative RP11-417E7.1 expression in CRC tissues with or without lymph node metastasis. N (-): CRC tissues without lymph node metastasis; N (+): CRC tissues with lymph node metastasis. **H**, qRT‒PCR analysis of relative RP11-417E7.1 expression in CRC tissues with or without distant metastasis. M (-): CRC tissues without distant metastasis; M (+): CRC tissues with distant metastasis. **I-J**, Kaplan‒Meier analysis of the disease-free survival and overall survival rates of CRC patients with high or low RP11-417E7.1 expression (from GEPIA2, *n* = 359). The data represent the findings from three independent experiments and are shown as the means ± SDs (*, *p* < 0.05; **, *p* < 0.01)

Even in early-stage T2 tumors (primary tumor invading the muscularis propria of the bowel wall but not into the subserosa or pericolic tissue), lymph node metastasis (25% of patients with T2 CRC) and distant metastasis (3.2% of patients with T2 CRC) are sometimes observed in clinical practice [18, 19]. We applied a lncRNA microarray analysis to primary tumor tissues from twelve patients with T2 CRC, including four patients without lymph node metastasis or distant metastasis (stage T2M0N0), four patients with ≥ 4 lymph node metastases but no distant metastasis (stage T2M0N2), and four patients with liver metastasis but no lymph node metastasis (stage T2M1N0), to identify the key lncRNAs that accelerate tumor metastasis in the relatively early stages of CRC. Compared with those in the T2M0N0 group, 196 differentially expressed lncRNAs (DElncRNAs; 154 upregulated and 42 downregulated) were identified (|logFC|≥ 1, *p* < 0.01; Fig. 1A) in the T2M0N2 group, and 457 DElncRNAs (292 upregulated and 165 downregulated) were identified (|logFC|≥ 1, *p* < 0.01; Fig. 1B) in the T2M1N0 group. After intersecting the two lists of DElncRNAs, we retrieved twelve hub metastasis-associated lncRNAs (eight upregulated and four downregulated, Fig. 1C). We focused on the eight upregulated lncRNAs, among which RP11-417E7.1 (transcript ID: ENST00000439703.1) showed significant fold changes in both the T2M0N2 group and T2M1N0 group (logFC = 2.98 and 2.67) (Figure S1A). Immunofluorescence and immunocytochemical staining confirmed that patients with T2M1N0 or T2M0N2 stage disease had higher levels of RP11-417E7.1 and higher expression of metastasis markers (Vimentin and N-cadherin) than patients with T2M0N0 stage disease (Figs. 1D). Specifically, we conducted Spearman’s analysis using 359 CRC samples (from TCGA-COAD and TCGA-READ datasets) through GEPIA2 (http://gepia2.cancer-pku.cn/#index). The analysis revealed positive correlations between RP11-417E7.1 expression and the expression of metastasis-related genes, including vimentin (*R* = 0.73, *p* < 0.01; Fig. 1E), N-cadherin (*R* = 0.66, *p* < 0.01; Fig. 1F), MMP-9 (*R* = 0.54, *p* < 0.01; Figure S1B), and Snail (*R* = 0.55, *p* < 0.01; Figure S1C). Similar correlations were found in other tumors (Figure S2A-G). These data suggest that RP11-417E7.1 is involved in CRC metastasis.

We further retrieved RP11-417E7.1 expression data from 80 CRC tissues and evaluated the potential correlation between RP11-417E7.1 expression and clinicopathological characteristics. qRT‒PCR analysis revealed that RP11-417E7.1 was significantly upregulated in N+ (with lymph node metastasis) or M+ (with distant metastasis) patients compared with N- (without lymph node metastasis) or M- (without distant metastasis) patients (Figs. 1G-H). Furthermore, we performed a survival analysis to investigate the association between RP11-417E7.1 expression and the prognosis of CRC patients using RNA-seq data and clinical information from the GEPIA2 database; 359 patients were included in our study cohort. Patients were divided into high and low RP11-417E7.1 expression groups based on the median RP11-417E7.1 expression level in CRC tissue samples. The Kaplan‒Meier (K‒M) analysis revealed that higher RP11-417E7.1 expression was associated with lower overall survival (OS) (*p* < 0.01; Fig. 1I) and disease-free survival (DFS) rates (*p* < 0.05; Fig. 1J). Similar prognostic results were found in other tumors (Figure S2H-M). These results indicate that RP11-417E7.1 may promote CRC metastasis and affect patient prognosis.

### RP11-417E7.1 enhances the metastatic capacity of CRC cells in vitro

**Fig. 2:**
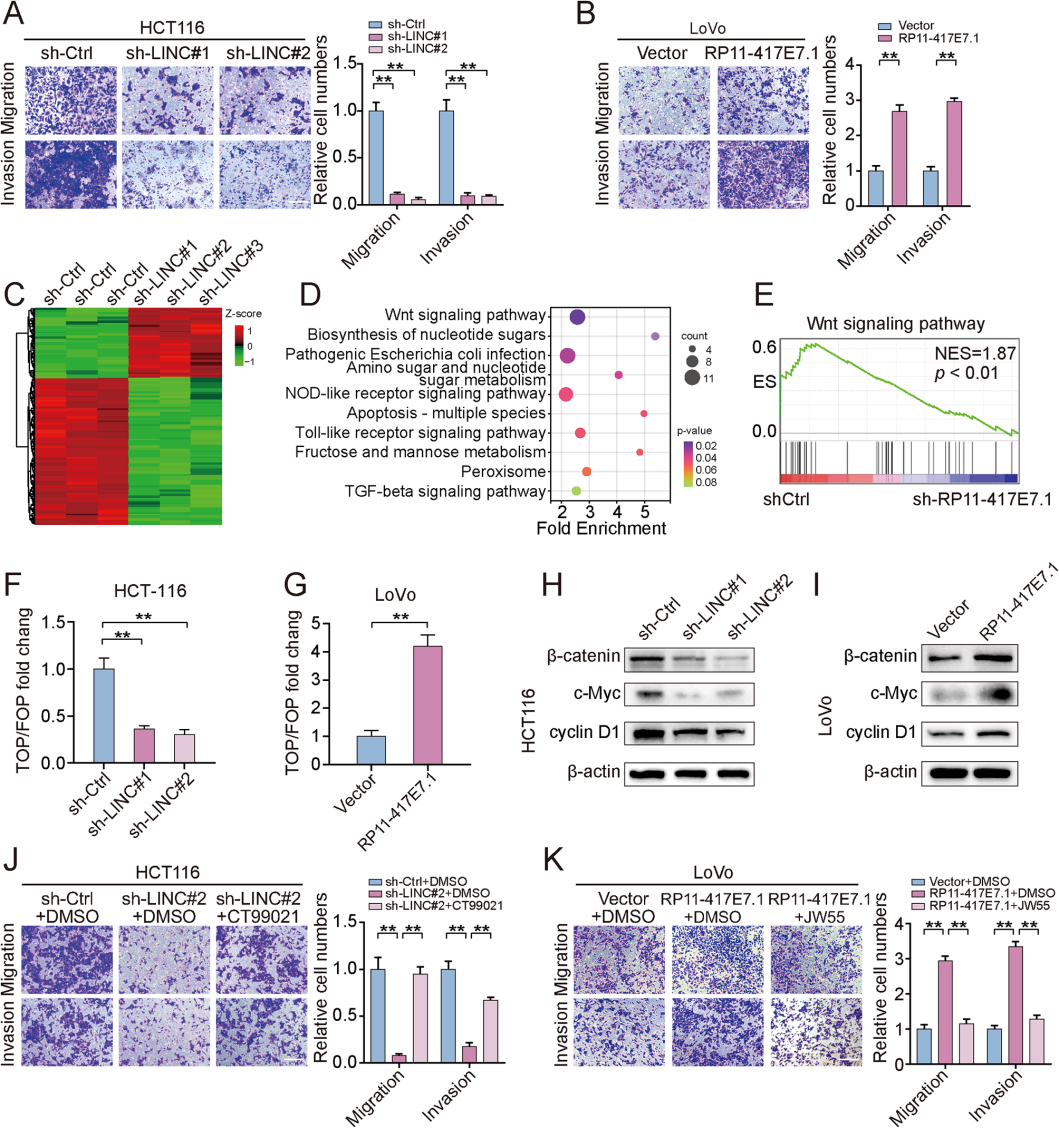
RP11-417E7.1 promotes CRC metastasis via the activation of Wnt/β-catenin signaling. **A-B**, Transwell assays showing the motility of RP11-417E7.1-depleted cells (A) or RP11-417E7.1-overexpressing cells (B) (left image). Histogram showing the results of the analysis of migrated cell counts (right image). Scale bar: 100 μm. **C**, Heatmap of differentially expressed genes after RP11-417E7.1 knockdown in HCT116 cells (*n* = 3). **D**, Bubble chart showing the KEGG analysis of differentially expressed genes in response to RP11-417E7.1 knockdown. **E**, GSEA showing that the genes that were differentially expressed after RP11-417E7.1 knockdown were enriched in the Wnt/β-catenin signaling gene set. **F-G**, TOP/FOP flash assay for the detection of Wnt/β-catenin pathway activity in RP11-417E7.1-knockdown (F) and RP11-417E7.1-overexpressing (G) CRC cells. **H**, Western blot analysis of β-catenin and downstream targets in the control and shRP11-417E7.1 groups. **I**, Western blot analysis of β-catenin and downstream targets in the control and RP11-417E7.1-overexpressing groups. **J**, Transwell assay showing the migratory and invasive potential of cells from the control, shRP11-417E7.1, and shRP11-417E7.1 + CT99021 groups. Scale bar: 100 μm. **K**, Transwell assays showing the migratory and invasive potential of cells from the control, RP11-417E7.1, and RP11-417E7.1 + JW55 groups. Scale bar: 100 μm. The data represent the findings from three independent experiments and are shown as the means ± SDs (*, *p* < 0.05; **, *p* < 0.01)

We first evaluated the expression of RP11-417E7.1 in normal intestinal epithelial FHC and CRC cell lines. The qRT‒PCR results showed that RP11-417E7.1 expression was relatively high in HCT116 and DLD1 cells and relatively low in LoVo cells (Figure S3A). Then, we established RP11-417E7.1-overexpressing CRC cell lines and RP11-417E7.1-silenced cell lines (Figure S3B-D). Transwell assays revealed that RP11-417E7.1 knockdown markedly decreased the migration and invasion of HCT116 and DLD1 cells (Figs. 2A and S3E), whereas RP11-417E7.1 overexpression significantly increased the motility of LoVo cells (Figs. 2B). As the EMT is an initial step in cancer cell dissemination, we assessed the expression of EMT-related proteins after manipulating RP11-417E7.1 expression in CRC cells. Silencing RP11-417E7.1 increased the expression of the epithelial marker E-cadherin and decreased the expression of the interstitial markers vimentin and N-cadherin (Figure S3F-G). RP11-417E7.1 overexpression resulted in opposite EMT-related protein expression patterns in LoVo cells (Figure S3H). Next, we conducted fibronectin cell adhesion assays to test the adhesion of CRC cells to the extracellular matrix (ECM). The knockdown of RP11-417E7.1 inhibited the cell–ECM adhesion of CRC cells (Figure S3I-J), whereas the overexpression of RP11-417E7.1 increased cell adhesion (Figure S3K). These data demonstrate that RP11-417E7.1 promotes the EMT, migration and invasion of CRC cells in vitro.

### RP11-417E7.1 promotes CRC metastasis via the activation of Wnt/β-catenin signaling

We conducted RNA-seq on RP11-417E7.1-silenced and control HCT116 cells to elucidate the mechanism by which RP11-417E7.1 affects CRC metastasis. RP11-417E7.1 depletion resulted in the upregulation of 158 genes and the downregulation of 334 genes (|FC|≥1.5, *p* < 0.01; Fig. 2C). Kyoto Encyclopedia of Genes and Genomes (KEGG) analysis and gene set enrichment analysis (GSEA) suggested that the differentially expressed genes (DEGs) were significantly enriched in the Wnt/β-catenin pathway (Figs. 2D-E). We then investigated whether RP11-417E7.1 is involved in the activation of Wnt/β-catenin signaling using a TOPflash reporter assay. Silencing of RP11-417E7.1 suppressed the activity of the β-catenin-dependent reporter (Figs. 2F), whereas overexpression of RP11-417E7.1 significantly increased the luciferase activity in CRC cells (Figs. 2G). Western blot assays further showed that β-catenin and downstream proteins (cyclin D1 and c-Myc) were downregulated upon RP11-417E7.1 depletion and upregulated upon RP11-417E7.1 overexpression (Figs. 2H-I and S3L).

We treated RP11-417E7.1-silenced CRC cells with the specific Wnt/β-catenin activator CT99021 (3 µM for 24 h) to determine whether RP11-417E7.1 promotes metastasis via Wnt/β-catenin signaling. CT99021 reversed the downregulation of β-catenin and EMT-related markers in RP11-417E7.1-depleted cells (Figure S3M). CT99021 also rescued the invasion and migration of RP11-417E7.1 knockdown cells (Figs. 2J). Moreover, the Wnt/β-catenin inhibitor JW55 (3 µM for 24 h) attenuated the protein expression of β-catenin and EMT-related markers, as well as the metastatic potential of CRC cells overexpressing RP11-417E7.1 (Figs. 2K and S3N). These results indicate that RP11-417E7.1 contributes to CRC metastasis by activating Wnt/β-catenin signaling.

### RP11-417E7.1 activates Wnt/β-catenin signaling by regulating THBS2 expression

**Fig. 3:**
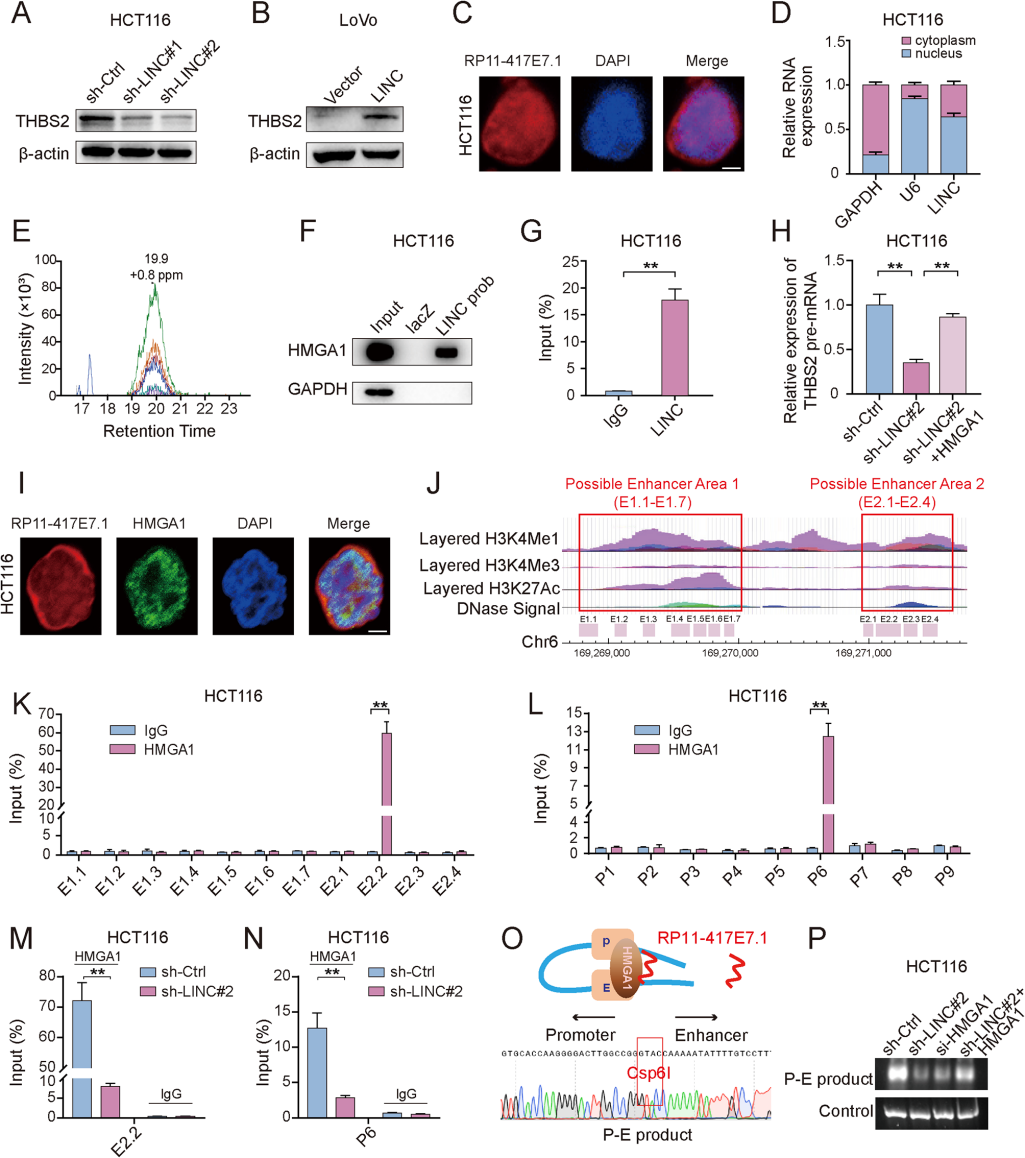
RP11-417E7.1 facilitates long-range promoter–enhancer interactions to increase THBS2 transcription by recruiting HMGA1. **A-B** Western blot analysis of THBS2 levels after RP11-417E7.1 silencing (A) or overexpression (B). **C**, RNA-FISH assay showing the localization of RP11-417E7.1 in HCT116 cells incubated with the RP11-417E7.1 probe (red), and nuclei were stained with DAPI (blue). Scale bar: 5 μm. **D**, qRT‒PCR analysis of the distribution of RP11-417E7.1, U6 and GAPDH in the cytoplasmic and nuclear fractions of CRC cells. U6 and GAPDH were used as the nuclear and cytoplasmic controls, respectively. **E**, Parallel reaction monitoring (PRM) assay showing the interaction between RP11-417E7.1 and HMGA1. **F**, Western blot analysis of HMGA1 protein expression after chromatin isolation by RNA purification (ChIRP) using RP11-417E7.1 probes. **G**, RNA immunoprecipitation (RIP) and qRT‒PCR showing the enrichment of RP11-417E7.1 after the immunoprecipitation of HMGA1. **H**, qRT‒PCR analysis of THBS2 pre-mRNA levels in RP11-417E7.1-silenced CRC cells transfected with the control plasmid or HMGA1 plasmid. **I**, Colocalization analysis with specific probes against RP11-417E7.1 (red) and a specific antibody against HMGA1 (green). Nuclei were stained with DAPI (blue). Scale bar: 5 μm. **J**, Chromatin immunoprecipitation sequencing (ChIP-seq) profiles of H3K4Me1, H3K4Me3, H3K27Ac and high DNase hypersensitivity at predicted enhancer-associated gene loci. The x-axis shows the locations on chromatin 6. The y-axis represents the reads per million (rpm) from ChIP-seq data. Potential enhancer areas are indicated with a red border. The red boxes indicate the ChIP‒qPCR primers targeting the potential THBS2 enhancers. **K**, ChIP‒qPCR results showing the enrichment of HMGA1 at the enhancer regions of THBS2 in HCT116 cells. **L**, ChIP‒qPCR results showing the enrichment of HMGA1 at the THBS2 promoter in HCT116 cells using primers for the promoter (P1‒P9). **M**, ChIP‒qPCR results showing changes in HMGA1 recruitment to the E2.2 region after RP11-417E7.1 knockdown in HCT116 cells. **N**, ChIP‒qPCR results showing changes in HMGA1 recruitment to the THBS2 promoter after RP11-417E7.1 knockdown. **O**, The upper schematic diagram shows how RP11-417E7.1 functions to sustain the chromatin loop for THBS2 expression. The bottom sequencing plot shows loop structure formation between the enhancer locus (15 kb upstream of THBS2) and the THBS2 promoter. **P**, Results of 3 C assays of chromatin loop products in the control, shRP11-417E7.1, siHMGA1, and shRP11-417E7.1 + HMGA1 groups. The data represent the findings from three independent experiments and are shown as the means ± SDs (*, *p* < 0.05; **, *p* < 0.01)

LncRNAs function mainly through the regulation of the expression of neighboring (*cis*-acting) or distant (*trans*-acting) protein-coding genes [20]. We then investigated whether RP11-417E7.1 mediated the expression of neighboring protein-coding genes. Spearman’s analysis revealed that RP11-417E7.1 expression was significantly positively correlated with the expression of the neighboring THBS2 gene in both our clinical samples (*R* = 0.63, *p* < 0.01) and TCGA dataset (*R* = 0.77, *p* < 0.01) (Figure S2G and S4A-B). Furthermore, the qRT‒PCR and Western blot results confirmed that RP11-417E7.1 could positively regulate the expression of THBS2 in CRC cells (Figs. 3A-B and S4C-F).

We then evaluated whether RP11-417E7.1 activated Wnt/β-catenin signaling through its neighboring gene THBS2. We overexpressed or silenced THBS2 in RP11-417E7.1-silenced or RP11-417E7.1-overexpressing cells. The decreases in β-catenin, c-Myc, and cyclin D1 levels caused by RP11-417E7.1 silencing were reversed when THBS2 was overexpressed (Figure S4G). Conversely, the increases in β-catenin, c-Myc, and cyclin D1 expression caused by RP11-417E7.1 overexpression were suppressed when THBS2 was depleted (Figure S4H). Furthermore, the addition of CT99021 restored β-catenin, c-Myc, and cyclin D1 protein expression in THBS2-silenced CRC cells (Figure S4I). JW55 treatment restored β-catenin, c-Myc, and cyclin D1 protein expression in THBS2-overexpressing CRC cells (Figure S4J). These results demonstrate that THBS2 is crucial for RP11-417E7.1-mediated Wnt/β-catenin signaling activation.

### The RP11-417E7.1-HMGA1 complex facilitates long-range promoter–enhancer interactions to increase THBS2 transcription

Next, we explored the potential mechanisms by which RP11-417E7.1 promoted THBS2 expression. Given that RP11-417E7.1 localizes to both the nucleus and cytoplasm (Figs. 3C-D), we first predicted the competing endogenous RNAs (ceRNAs) of THBS2 using the StarBase website, but RP11-417E7.1 is not a ceRNA of THBS2 (data not shown). RNA‒protein interactions are important strategies by which lncRNAs influence gene expression [10]. We further examined proteins that could interact with RP11-417E7.1 to drive THBS2 transcription. The chromatin isolation by RNA purification (ChIRP) assay combined with mass spectrometry (MS) data showed that HMGA1 could specifically bind RP11-417E7.1 (Additional file 1). The results of parallel reaction monitoring (PRM), ChIRP combined with Western blot, and RIP combined with qRT‒PCR further confirmed the interaction between RP11-417E7.1 and HMGA1 (Figs. 3E-G and S5A-B). In addition, RP11-417E7.1 knockdown did not affect HMGA1 expression (Figure S5C). Immunofluorescence colocalization results showed that RP11-417E7.1 and HMGA1 colocalized in the nucleus of CRC cells (Figs. 3I). The overexpression of HMGA1 in RP11-417E7.1-depleted cells increased THBS2 mRNA and pre-mRNA expression (Figs. 3H and S5D). In contrast, knockdown of HMGA1 in RP11-417E7.1-overexpressing cells suppressed THBS2 mRNA and pre-mRNA expression (Figure S5E-F). These data suggest that HMGA1 mediates the regulatory effect of RP11-417E7.1 on THBS2 transcription.

Interestingly, after knockdown of RP11-417E7.1, a promoter–reporter assay showed that THBS2 promoter activity was not affected (Figure S5G). As HMGA1 has been reported to mediate direct interactions between DNA sequences [21], we speculated that RP11-417E7.1 possibly promotes THBS2 transcription by maintaining enhancer–promoter interactions with HMGA1. Active enhancers are characterized by the enrichment of histone posttranslational modifications such as H3K4Me1 and H3K27Ac, as well as DNase hypersensitivity [22, 23]. Based on chromatin immunoprecipitation followed by sequencing (ChIP–seq) data from UCSC (http://genome.ucsc.edu/), we identified two potential enhancer areas approximately 12 kb (E1 area) and 15 kb (E2 area) upstream of THBS2 based on the high enrichment of H3K4Me1 and H3K27Ac and high DNase hypersensitivity (Figs. 3J). We designed 11 pairs of PCR primers that covered the predicted enhancer regions (E1.1-E1.7 and E2.1-E2.4) to identify specific binding sites. The ChIRP–qPCR results revealed that this nuclear lncRNA bound to enhancer region E2.2 (Figure S6A-B), and the ChIP‒qPCR results showed that HMGA1 was also specifically enriched in enhancer region 2.2 (Figs. 3K and S6C). Notably, the enrichment of HMGA1 was inhibited in response to RP11-417E7.1 knockdown (Figs. 3M and S6D). We designed nine pairs of PCR primers that covered the promoter region (Figure S6E) and then performed ChIRP–qPCR and ChIP‒qPCR assays to test whether the RP11-417E7.1-HMGA1 complex was recruited to the genomic sequence of the THBS2 promoter. RP11-417E7.1 and HMGA1 bound to the THBS2 promoter region (P6) (Figs. 3L and S6F-H). Knockdown of RP11-417E7.1 led to a drastic reduction in HMGA1 expression in the THBS2 promoter region (Figs. 3N and S6I). Next, chromosome conformation capture (3 C) combined with qPCR and sequencing confirmed the formation of a loop structure between the enhancer locus (15 kb upstream of THBS2) and the THBS2 promoter (Figs. 3O). Treatment with siHMGA1 or shRP11-417E7.1 abolished 3 C product formation, but HMGA1 overexpression increased the level of the 3 C product in shRP11-417E7.1 cells (Figs. 3P and S6J). Collectively, our findings show that RP11-417E7.1 recruits HMGA1 to both the enhancer and promoter regions of THBS2, thus promoting the formation of a specific chromatin loop and enhancing THBS2 transcription.

### THBS2 associates with YWHAZ to activate Wnt/β-catenin signaling by inhibiting ubiquitylation-mediated β-catenin degradation

**Fig. 4:**
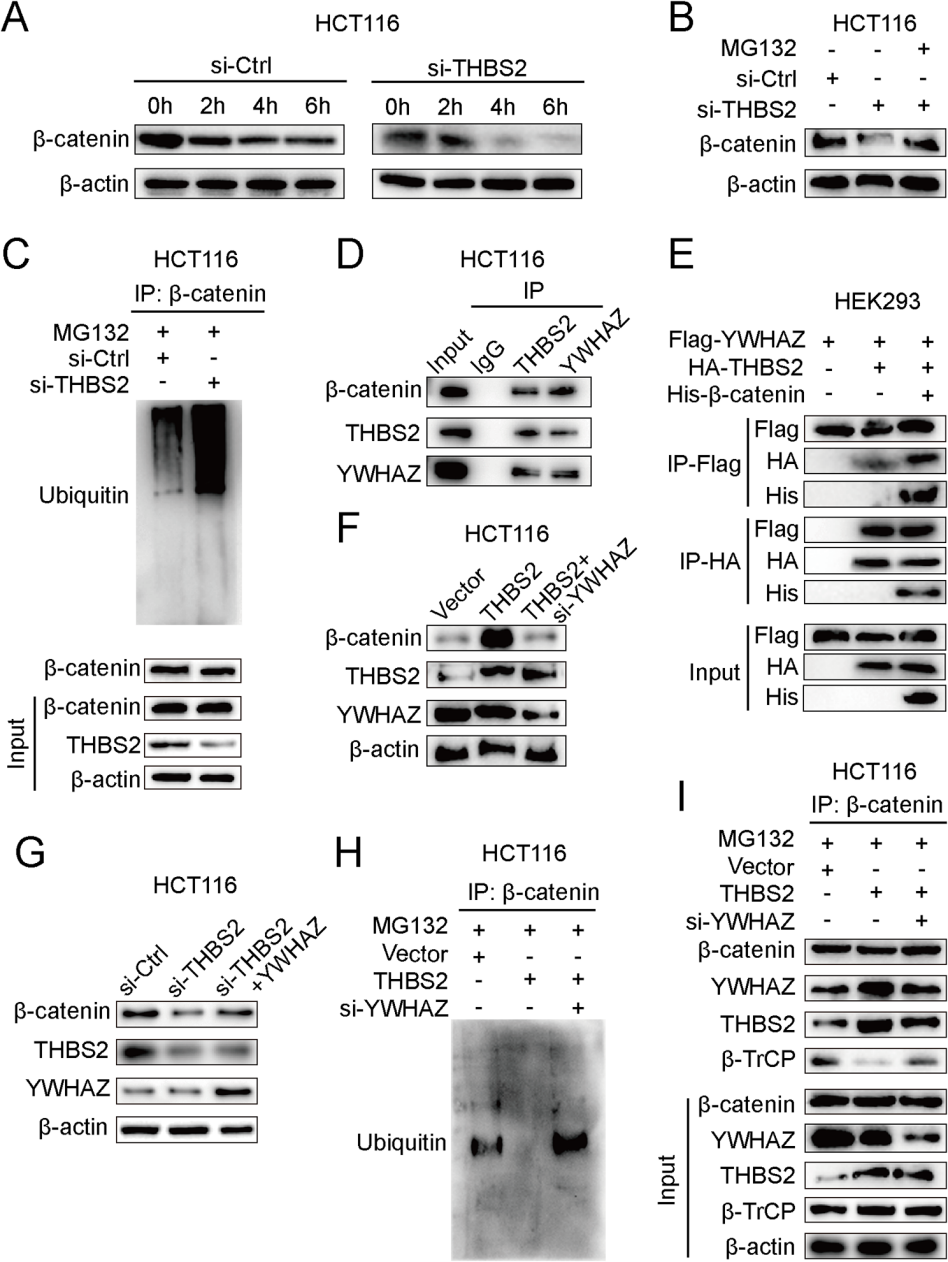
THBS2 interacts with YWHAZ to inhibit ubiquitylation-mediated β-catenin degradation. **A**, Western blot analysis of β-catenin levels after cycloheximide (CHX) treatment at the indicated time points (25 µg/ml). **B**, Western blot assays of β-catenin levels in cells treated with CHX alone or CHX plus MG-132 (20 µΜ) for 4 h. **C**, Immunoprecipitation and western blotting were used to detect β-catenin ubiquitination in the control and si-THBS2 groups. **D-E**, Western blot analysis of the interaction between THBS2, YWHAZ, and β-catenin in CRC and HEK293 cells. **F**, Western blot analysis of β-catenin levels in THBS2-overexpressing cells transfected with si-YWHAZ or siRNA ctrl. **G**, Western blot analysis of β-catenin levels in THBS2-depleted HCT116 cells treated with YWHAZ overexpression or control plasmid. **H-I**, Extracts of HCT116 cells were subjected to immunoprecipitation using a β-catenin antibody followed by Western blot assays using antibodies against ubiquitin (H) and ubiquitination-related proteins (I) in THBS2-overexpressing HCT116 cells transfected with si-YWHAZ or siRNA ctrl. The data represent the findings from three independent experiments and are shown as the means ± SDs (*, *p* < 0.05; **, *p* < 0.01)

The mechanism by which THBS2 activates Wnt/β-catenin signaling is unknown. As THBS2 can positively regulate the expression of the β-catenin protein, but not mRNA (Figure S6K), we examined the stability of the β-catenin protein in response to THBS2 knockdown. Intriguingly, THBS2 knockdown led to more rapid degradation of β-catenin (Figs. 4A). Since the stability of β-catenin is regulated mainly by the ubiquitination–proteasome degradation pathway [24], we examined the β-catenin levels in various groups in the presence of the proteasome inhibitor MG132 and found that β-catenin degradation could be prevented in THBS2-silenced CRC cells (Figs. 4B). Consistent with this finding, the depletion of THBS2 increased the level of β-catenin ubiquitylation (Figs. 4C). We used the STRING interactive database (https://version-10-5.string-db.org/cgi/input.pl) to predict proteins that interact with THBS2 and to explore the molecular mechanism by which THBS2 regulates ubiquitylation. Among these proteins, YWHAZ was found to potentially interact significantly with THBS2 (Figure S6L). Previous studies have shown that YWHAZ can block β-catenin ubiquitylation by competing with the E3 ligase β-TrCP to interact with β-catenin [25]. We examined whether YWHAZ participates in the THBS2-mediated degradation of β-catenin. Coimmunoprecipitation (Co-IP) assays showed that THBS2 bound to YWHAZ and β-catenin in CRC cells (Figs. 4D). The interaction between THBS2, YWHAZ, and β-catenin was further confirmed by co-IP when the corresponding labeled proteins were coexpressed in HEK293 cells (Figs. 4E). YWHAZ knockdown suppressed the THBS2-mediated upregulation of β-catenin (Figs. 4F). Conversely, YWHAZ overexpression reversed the reduction in β-catenin expression mediated by THBS2 depletion (Figs. 4G). The results of the immunoprecipitation assay showed that YWHAZ suppression significantly increased β-catenin ubiquitylation in THBS2-overexpressing CRC cells (Figs. 4H). We examined the interaction between β-TrCP and β-catenin by co-IP to determine whether the E3 ligase β-TrCP is involved in THBS2-mediated reduction in β-catenin degradation. THBS2 overexpression suppressed the binding between β-TrCP and β-catenin, whereas YWHAZ silencing strengthened this binding in THBS2-overexpressing cells (Figs. 4I). These findings suggest that THBS2 recruits YWHAZ to impede the ubiquitylation-mediated degradation of β-catenin by competitively inhibiting its binding to β-TrCP.

### Exosomal THBS2 induces M2 polarization of TAMs by activating Wnt/β-catenin signaling

Several studies have mentioned that THBS2 expression is also associated with remodeling of the tumor immune microenvironment [26, 27]. We evaluated the correlation of THBS2 level with the proportion of tumor-infiltrating immune cells as well as immune response in TCGA CRC datasets using the CIBERSORT algorithm and Spearman’s analysis [28]. The results revealed that THBS2 expression was associated with immunosuppressive response and had the strong correlations with the immunosuppressive immune cells, especially M2 macrophages (*R* = 0.48, *p* < 0.01) (Figure S7A-G), suggesting a potential role for THBS2 in promoting M2 macrophage polarization. A recent study showed that THBS2 can be released into the TME through exosome secretion in lung adenocarcinoma [29]. Therefore, we hypothesized that RP11-417E7.1/THBS2 signaling may promote tumor cell secretion of THBS2 into the TME in the form of exosomes, thereby inducing M2 macrophage polarization in CRC.

**Fig. 5:**
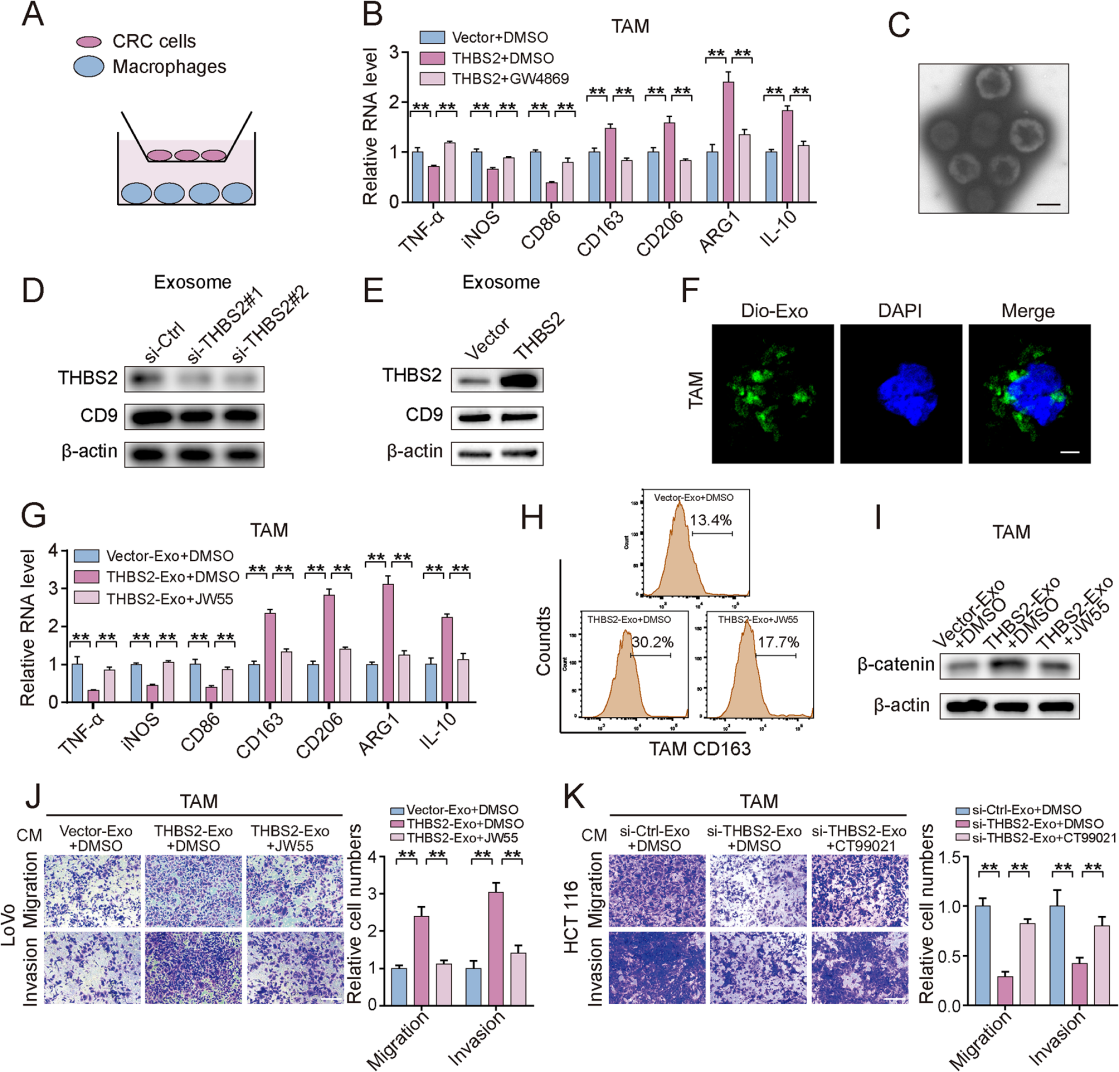
Exosomal THBS2 induces the M2 polarization of TAMs by activating Wnt/β-catenin signaling. **A**, Schematic diagram of a coculture system for macrophages and CRC cells. **B**, Macrophages were cocultured with THBS2-overexpressing cells with or without GW4869 (10 µM). qRT‒PCR was used to measure the expression of M1 and M2 markers in macrophages. **C**, TEM images of the exosomes extracted from LoVo cells. Scale bar: 100 nm. **D-E**, Western blot analysis of exosomal THBS2 expression in THBS2-knockdown HCT116 cells (D) and THBS2-overexpressing LoVo cells (E). CD9 was used as an exosome-positive marker. **F**, Exosomes isolated from CRC cells were pretreated with DiO and applied to macrophages. Fluorescence microscopy indicated the process by which LoVo cell-derived exosomes were transferred to macrophages. LoVo cell-derived exosomes were labeled with DiO (green). Nuclei were stained with DAPI (blue). Scale bar: 10 μm. **G-I**, Exosomes (10 µg/ml) isolated from the supernatants of CRC cells treated with the THBS2 plasmid or control plasmid were added to macrophages. Twenty-four hours after exosome treatment, macrophages were treated with or without JW55. Then, qRT‒PCR was used to detect the expression of M1 and M2 markers (G); flow cytometry analysis was used to detect the counts of CD163 subpopulations (H); and Western blotting was used to detect the expression of the β-catenin protein (I). **J-K**, Transwell assays showing the migratory and invasive potential of CRC cells cultured in conditioned media from exosome-treated TAMs. Scale bar: 100 μm. The data represent the findings from three independent experiments and are shown as the means ± SDs (*, *p* < 0.05; **, *p* < 0.01)

We constructed a transwell coculture system in which CRC cells and PMA-induced THP-1 macrophages were incubated (Figs. 5A). The qRT‒PCR results showed that THBS2 overexpression in CRC cells led to the downregulation of M1 biomarkers (iNOS, TNF-α, and CD86) and the significant upregulation of M2 biomarkers (CD163, CD206, ARG1 and IL-10) in macrophages (Figs. 5B). Interestingly, these effects were counteracted by GW4869 (an exosome inhibitor) (Figs. 5B). We then monitored exosomes derived from CRC cells using transmission electron microscopy and confirmed that the exosomes were approximately 100 nm in diameter (Figs. 5C). Western blot results showed that THBS2 knockdown resulted in the downregulation of THBS2 within exosomes, while THBS2 overexpression led to an increase in exosomal THBS2 levels (Figs. 5D-E). In addition, CRC-derived exosomes labeled with DiO were collected and used to treat macrophages. DiO fluorescence in macrophages indicated the role of exosomes in CRC cell–macrophage crosstalk (Figs. 5F). These results suggest that THBS2-containing exosomes derived from CRC cells can induce macrophage polarization toward the M2 phenotype.

Activation of Wnt/β-catenin signaling in macrophages is known to promote M2 polarization [30]. We collected THBS2-overexpressing exosomes from CRC cells with THBS2 overexpression to treat macrophages and determine whether exosomal THBS2 promotes M2 polarization of macrophages via Wnt/β-catenin signaling. As expected, THBS2-overexpressing exosomes enhanced β-catenin protein expression and promoted the M2 polarization of macrophages, while JW55 treatment of macrophages abrogated these changes (Figs. 5G-I). In addition, exosomes derived from si-THBS2 CRC cells had the opposite effect (Figure S8A-B). These results indicate that exosomal THBS2 activates Wnt/β-catenin signaling in macrophages, thus inducing M2 macrophage polarization.

We treated macrophages with exosomes isolated from THBS2-overexpressing, THBS2-depleted, or control cells to investigate the effect of M2 macrophage polarization mediated by exosomal THBS2 on CRC cell metastasis. Then, we collected the conditioned medium of educated macrophages and used it to treat CRC cells. Macrophages treated with exosomes isolated from the THBS2-overexpressing groups showed significantly enhanced cell migration and invasion (Figs. 5J). Furthermore, macrophages treated with exosomes isolated from the si-THBS2 groups exhibited suppressed CRC cell migration and invasion (Figs. 5K). These data suggest that exosomal THBS2 derived from CRC cells enhances the M2 polarization of TAMs and subsequently accelerates CRC metastasis.

## The RP11-417E7.1/THBS2/β-catenin axis promotes CRC metastasis and M2 macrophage infiltration in vivo

**Fig. 6:**
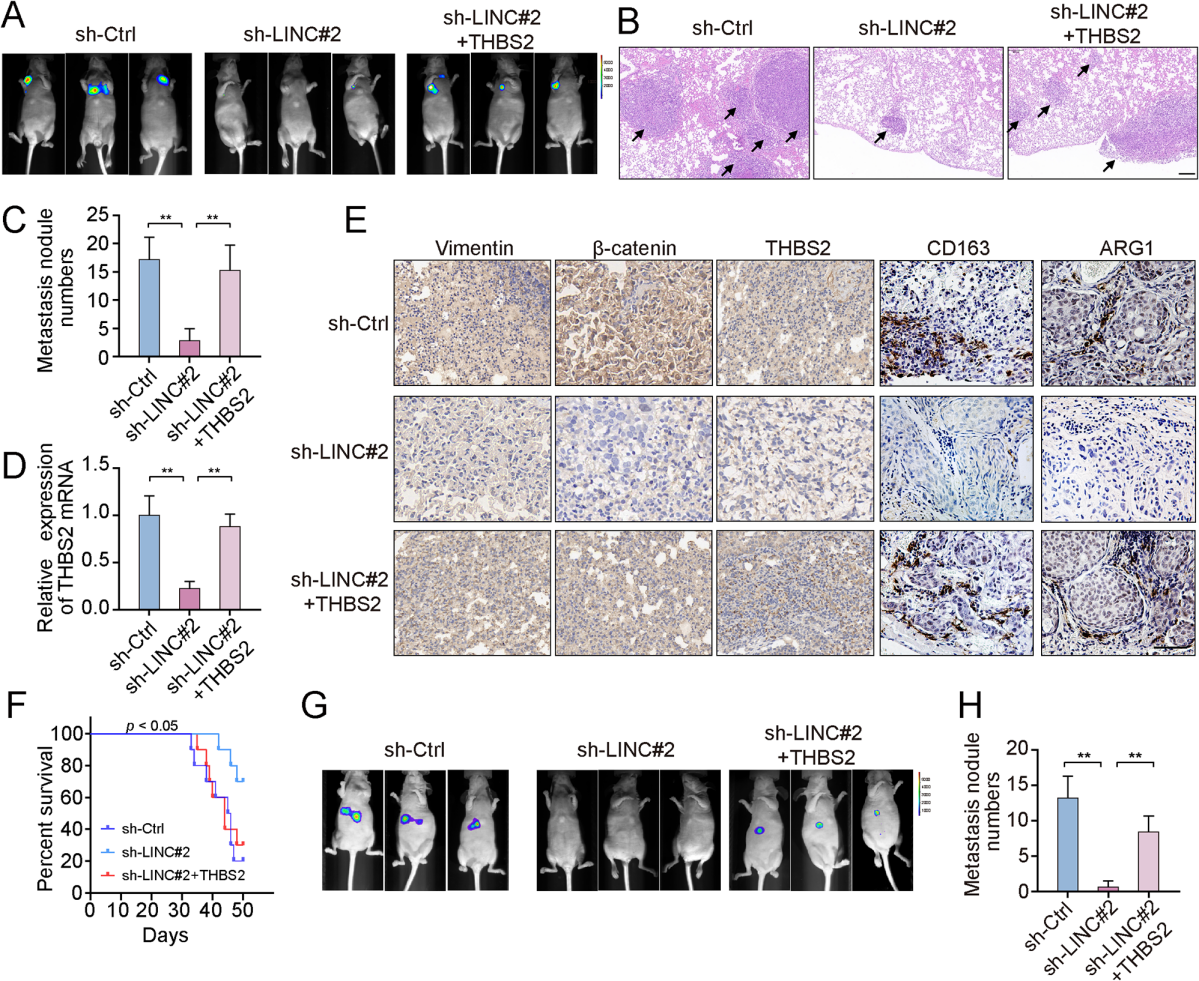
The RP11-417E7.1/THBS2 signaling pathway promotes CRC metastasis in vivo. **A**, Representative bioluminescent images of lung metastasis acquired through IVIS five weeks after infection from the control (*n* = 5), shRP11-417E7.1 (*n* = 5), and shRP11-417E7.1 + THBS2 groups (*n* = 5). **B**, HE staining of lung tissue sections showing metastases in each group. The arrows point to the lung nodules. Scale bar: 200 μm. **C**, The number of lung metastatic nodules in the tested mice. **D**, qRT‒PCR analysis of THBS2 levels in the control, shRP11-417E7.1, and shRP11-417E7.1 + THBS2 groups. **E**, IHC staining for THBS2, β-catenin, Vimentin, CD163, and ARG1 in the indicated groups is shown (×400 magnification). Scale bar: 50 μm. **F**, Kaplan‒Meier analysis showing the survival period of the mice in each group (*n* = 10). **G**, Representative bioluminescent images of metastases acquired through IVIS six weeks after injecting HCT116 cells into the spleens (*n* = 5). **H**, The number of liver metastatic nodules in the tested mice six weeks after injecting HCT116 cells into the spleens (*n* = 5). The data represent the findings from three independent experiments and are shown as the means ± SDs (*, *p* < 0.05; **, *p* < 0.01)

We established a lung metastasis mouse model by injecting processed CRC cells into the tail vein to assess the role of RP11-417E7.1 in CRC metastasis in vivo. As shown in Fig. 6A, five weeks after the injection, the bioluminescence signals in the shRP11-417E7.1 group were weaker than those in the control group. Consistently, the number and size of lung metastatic foci decreased upon RP11-417E7.1 knockdown (Figs. 6B-C). Notably, the impairment of metastasis caused by RP11-417E7.1 depletion was reversed by THBS2 overexpression (Figs. 6A-C). Furthermore, RP11-417E7.1 depletion downregulated the expression of Vimentin, THBS2, β-catenin, and the M2 macrophage markers CD163 and ARG1, while THBS2 overexpression restored their expression (Figs. 6D-E). Moreover, the silencing of RP11-417E7.1 markedly extended mouse survival (*p* < 0.05), while the overexpression of THBS2 counteracted this effect (Figs. 6F).

We also established a liver metastasis model by injecting HCT116 cells into the spleens. Six weeks after the injection, a significant reduction in liver metastasis was observed following RP11-417E7.1 knockdown, whereas THBS2 overexpression counteracted the inhibitory effect of shRP11-417E7.1 on tumor metastasis (Figs. 6G-H). These results support that RP11-417E7.1 promotes CRC metastasis by activating the THBS2/β-catenin pathway and increasing M2 macrophage infiltration in vivo.

### Netropsin inhibits CRC metastasis by blocking the effect of the RP11-417E7.1-HMGA1 complex on THBS2 transcription

**Fig. 7:**
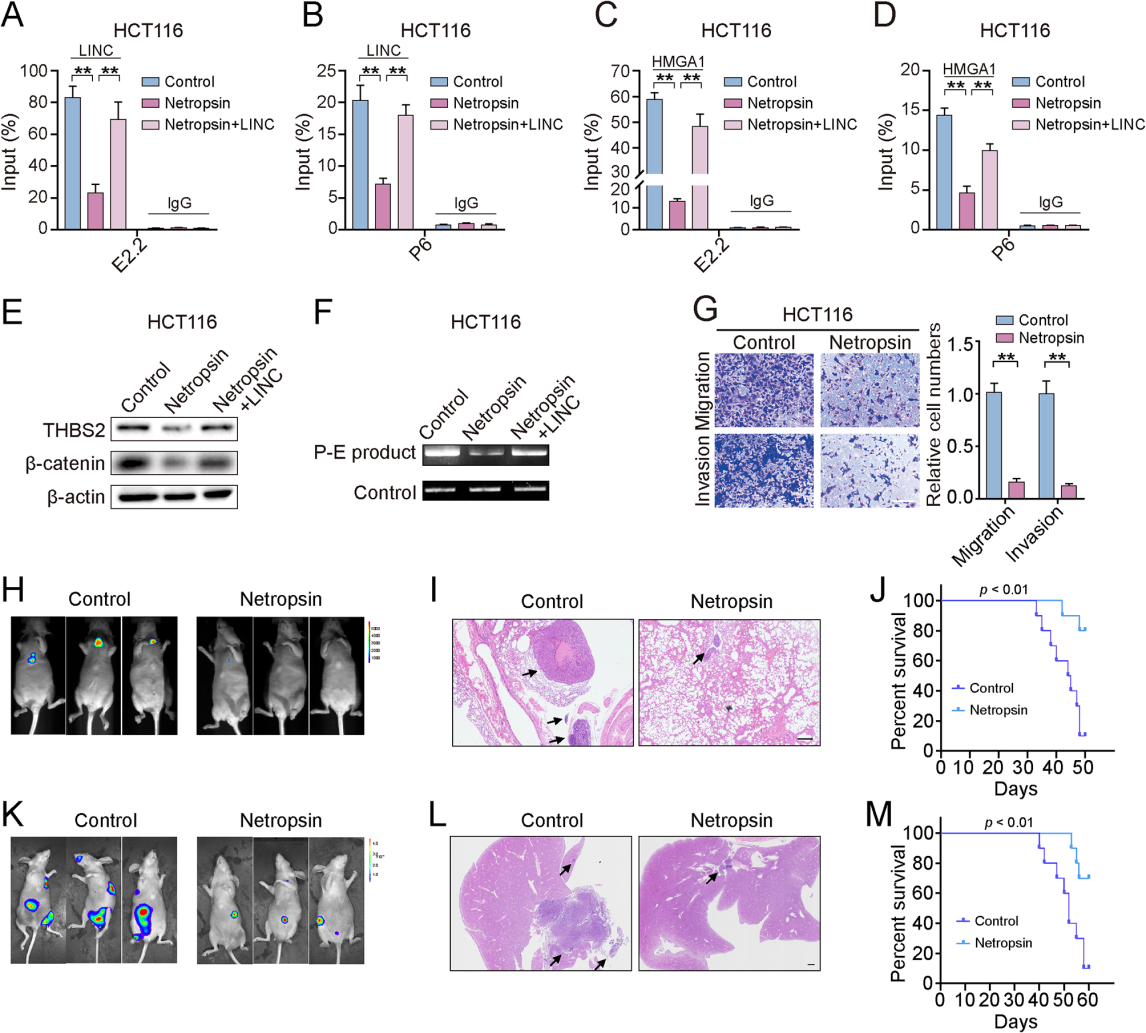
Netropsin inhibits CRC metastasis by blocking the effect of RP11-417E7.1. **A**, ChIRP-qPCR showed RP11-417E7.1 recruitment to the E2.2 region in HCT116 cells from the control, netropsin, and netropsin + RP11-417E7.1 groups. **B**, ChIRP–qPCR showed RP11-417E7.1 recruitment to the THBS2 promoter in the indicated groups. **C**, ChIP‒qPCR showing HMGA1 recruitment to the E2.2 region in HCT116 cells from the control, netropsin, and netropsin + RP11-417E7.1 groups. **D**, ChIP‒qPCR analysis of HMGA1 recruitment to the THBS2 promoter in the indicated groups. **E**, Western blot analysis of THBS2 and β-catenin levels in the indicated groups. **F**, Results of 3 C assays of chromatin loop products in the control, netropsin, and netropsin + RP11-417E7.1 groups. **G**, Transwell assays showing changes in the migratory and invasive potential of CRC cells after netropsin treatment. Scale bar: 100 μm. **H**, Representative bioluminescent images of lung metastases acquired through IVIS five weeks after HCT116 cell injection in the control (*n* = 5) and netropsin treatment groups (*n* = 5). **I**, HE staining of lung tissue sections showing metastases in each group. The arrows point to the lung nodule. Scale bar: 200 μm. **J**, Kaplan‒Meier analysis showing the survival period of mice in each lung metastasis group (*n* = 10). **K**, Representative bioluminescent images of orthotopic tumors and liver metastases acquired through IVIS six weeks after HCT116 cell injection of an orthotopic mouse cancer model for each group (*n* = 5). **L**, HE staining of liver tissue sections showing metastases in each group. The arrows point to the lung nodule. Scale bar: 500 μm. **M**, Kaplan‒Meier analysis showing the survival period of mice in each orthotopic model group (*n* = 10). The data represent the findings from three independent experiments and are shown as the means ± SDs (*, *p* < 0.05; **, *p* < 0.01)

The antibiotic netropsin is a potential antineoplastic drug that competes with HMGA1 for binding to DNA sequences [31]. Indeed, the addition of netropsin to cultures for two days inhibited the enrichment of HMGA1 and RP11-417E7.1 at the promoter and enhancer regions of THBS2 in HCT116 cells, while RP11-417E7.1 overexpression restored the enrichment of HMGA1 and RP11-417E7.1 in netropsin-treated cells (Figs. 7A-D). Accordingly, the expression of THBS2 and β-catenin was significantly downregulated by netropsin, but this effect was reversed by the overexpression of RP11-417E7.1 (Figs. 7E). We further explored the effect of netropsin on RP11-417E7.1-mediated chromatin looping. The 3 C combined with qPCR analysis verified that netropsin reduced chromatin looping in CRC cells, but RP11-417E7.1 overexpression abolished the inhibitory effect of netropsin on chromatin looping (Figs. 7F). These results indicate that netropsin impedes RP11-417E7.1-mediated HMGA1 recruitment to the promoter and enhancer of THBS2, thus inhibiting chromatin looping and THBS2 transcription.

The effect of netropsin on CRC metastasis remains unknown. We next examined whether netropsin could inhibit CRC metastasis in vivo and in vitro. Transwell assays showed that netropsin treatment significantly reduced the migration and invasion of CRC cells (Figs. 7G). Moreover, in the lung metastasis mouse model, the intraperitoneal administration of netropsin led to weaker bioluminescent signals in the lungs than in the control group (Figs. 7H). HE staining further confirmed that the number and size of metastatic lung nodules decreased following netropsin treatment (Figs. 7I). We then evaluated the effect of netropsin on the survival of mice harboring lung metastases. The K‒M plot showed a significant prolongation of the OS of the mice treated with netropsin (*p* < 0.01) (Figs. 7J). We further established an orthotopic model through the inoculation of HCT116 cells in the cecum to simulate the progression from the primary tumor to metastatic CRC. Intraperitoneal administration of netropsin significantly decreased intra-abdominal and liver metastases (Figs. 7K-L) and improved the OS of the mice (*p* < 0.01) (Figs. 7M). These data reveal that netropsin treatment can effectively suppress CRC metastasis.

## Discussion

Metastatic disease accounts for the vast majority of cancer-associated deaths. Emerging evidence has shown the important roles of lncRNAs in cancer progression, especially their effects on cancer metastasis [32]. In our study, we found that the expression of a metastasis-related lncRNA, RP11-417E7.1, was significantly elevated in early T2 CRC tissues with lymph node metastasis or distant metastasis. High expression of RP11-417E7.1 indicated a poor prognosis for CRC patients. Depletion of RP11-417E7.1 markedly inhibited CRC metastasis in vivo and in vitro. These data suggest that RP11-417E7.1 might serve as a novel biomarker or therapeutic target for CRC metastasis.

LncRNAs can be categorized into *cis*-acting or *trans*-acting lncRNAs based on the local or distant regulation of gene expression [33]. *Cis*-acting lncRNAs regulate nearby gene expression on the same chromosome from which they are transcribed, whereas *trans*-acting lncRNAs regulate gene transcription on different chromosomes [10]. By exploring the mechanism by which RP11-417E7.1 activates the Wnt pathway, we discovered a significant correlation between the expression of RP11-417E7.1 and its neighboring gene THBS2 based on RNA sequencing data from CRC samples. Further study revealed that THBS2 mediated the effects of RP11-417E7.1 on Wnt pathway activation, suggesting a *cis*-regulatory function of RP11-417E7.1. We analyzed RP11-417E7.1-interacting proteins via ChIRP-MS to understand the mechanism by which RP11-417E7.1 modulates the expression of nearby genes, which led to the identification of 20 binding proteins, including HMGA1. HMGA1 belongs to the high mobility group (HMG) protein family characterized by the presence of three AT-hook DNA binding motifs that preferentially bind to the minor groove of A/T-rich B-form DNA sequences and maintain a more open DNA structure, which facilitates gene transcription [34]. In our study, we first measured THBS2 promoter activity upon RP11-417E7.1 knockdown, and no change in promoter activity was detected in the reporter assay. These results suggest that the mere absence of the promoter region is not sufficient to explain the effect of the RP11-417E7.1/HMGA1 complex on THBS2 transcription. Studies have reported that HMGA1, which is involved in enhanceosome formation, binds to A/T-rich DNA sequences in gene promoter and enhancer regions, regulating gene-specific transcription [35, 36]. We localized the enhancer region of THBS2 based on the ChIP-seq data of histone posttranslational modifications. ChIRP–qPCR confirmed that RP11-417E7.1 and HMGA1 bound to enhancer region E2.2 and the promoter region of THBS2, and 3 C combined with qPCR showed that RP11-417E7.1 recruited HMGA1 to facilitate enhancer–promoter loop formation. This study demonstrates that the lncRNA RP11-417E7.1 is involved in sustaining a specific enhancer–promoter looping structure, controlling downstream gene expression. Currently, very few studies have been conducted on lncRNAs that participate in chromatin looping. In lung cancer, the lncRNA MYMLR was shown to play a role in tumor progression by establishing an enhancer–promoter loop and activating MYC transcription [37]. However, a variety of novel lncRNAs involved in maintaining chromosomal structure still need to be discovered to address the underlying regulatory mechanisms involved.

The precise mechanism by which THBS2 activates the Wnt/β-catenin pathway remains unknown. Here, we report for the first time that YWHAZ bridges the link between THBS2 and β-catenin stability. YWHAZ, a member of the 14-3-3 protein family, is involved in many signal transduction pathways and plays an important role in tumor progression [38]. YWHAZ has been reported to stabilize β-catenin by binding competitively with β-TrCP binding sites to protect β-catenin from ubiquitination-dependent degradation in lung cancer [25]. Based on the above finding that YWHAZ regulates β-catenin stability, our findings revealed that THBS2 activates the Wnt/β-catenin pathway by recruiting YWHAZ to maintain β-catenin stability.

TAMs that can polarize to the M1 and M2 phenotypes are the most abundant immune cells in the TME. Tumor-infiltrating M2 TAMs directly promote cancer growth and metastasis by secreting regulatory cytokines, including IL-10 and ARG-1, whereas M1 TAMs have the opposite effects [39]. Interestingly, we found that THBS2 expression was positively correlated with the expression of M2-type TAM biomarkers. Further validation confirmed that THBS2 overexpression in CRC cells promoted the M2-like polarization of cocultured TAMs. These findings suggest that CRC cell secretion mediated by THBS2 may regulate the polarization of macrophages. A wide variety of molecules, such as cytokines, chemokines, exosomes and metabolites, can be secreted by tumor cells. Among these, cancer cell-secreted exosomes, which encapsulate and thus transmit biomolecules such as proteins to recipient cells, are pivotal mediators of intercellular communication within the TME [40]. A recent study reported that tumor-derived exosomes transmit CXCL14 to stimulate TAM M2 polarization through the activation of the NF-κB pathway [41]. Here, we discovered that THBS2 can be secreted by tumor cells into culture media through exosomes and that exosomal THBS2 can be transported into macrophages and induce M2 polarization by activating the Wnt signaling pathway. In addition to the function of THBS2 in CRC cells, our study revealed a dual function of THBS2 in regulating CRC metastasis: one function is to activate the intrinsic Wnt pathway in CRC cells to trigger distant metastasis, and the other is to induce TAM M2 polarization for a prometastatic TME after transmission in the form of exosomes.

Netropsin is one of the first discovered ligands that selectively binds to the minor groove of DNA and prevents HMGA1 from binding to DNA [42, 43]. It was previously reported to have antiviral activity and was subsequently shown to have cytostatic activity against tumor cell lines [42, 44]. For example, netropsin reduced the expression of cdc25A by suppressing its promoter activity and inhibited intracranial medulloblastoma cell growth both in vitro and in vivo [42]. However, the effects of netropsin on CRC remain unknown. Our study indicated that netropsin can competitively inhibit the binding of the RP11-417E7.1-HMGA1 complex to DNA, preventing CRC metastasis both in vivo and in vitro. At present, research on netropsin has focused mainly on animals or at the cellular level. Several limited in vivo studies did not observe any significant toxic side effects of netropsin on mice [42, 45]. However, comprehensive preclinical studies are needed to evaluate the safety of netropsin before its clinical application. In conclusion, this study revealed that the lncRNA RP11-417E7.1/THBS2 signaling pathway facilitates CRC metastasis. Notably, treatment with netropsin effectively disrupted this signaling pathway, resulting in the suppression of CRC metastasis. Therefore, this study elucidates the potential mechanism of RP11-417E7.1/THBS2 signaling, providing an effective and promising therapeutic strategy for CRC patients, especially those expressing a high level of RP11-417E7.1.

## Conclusions

**Fig. 8:**
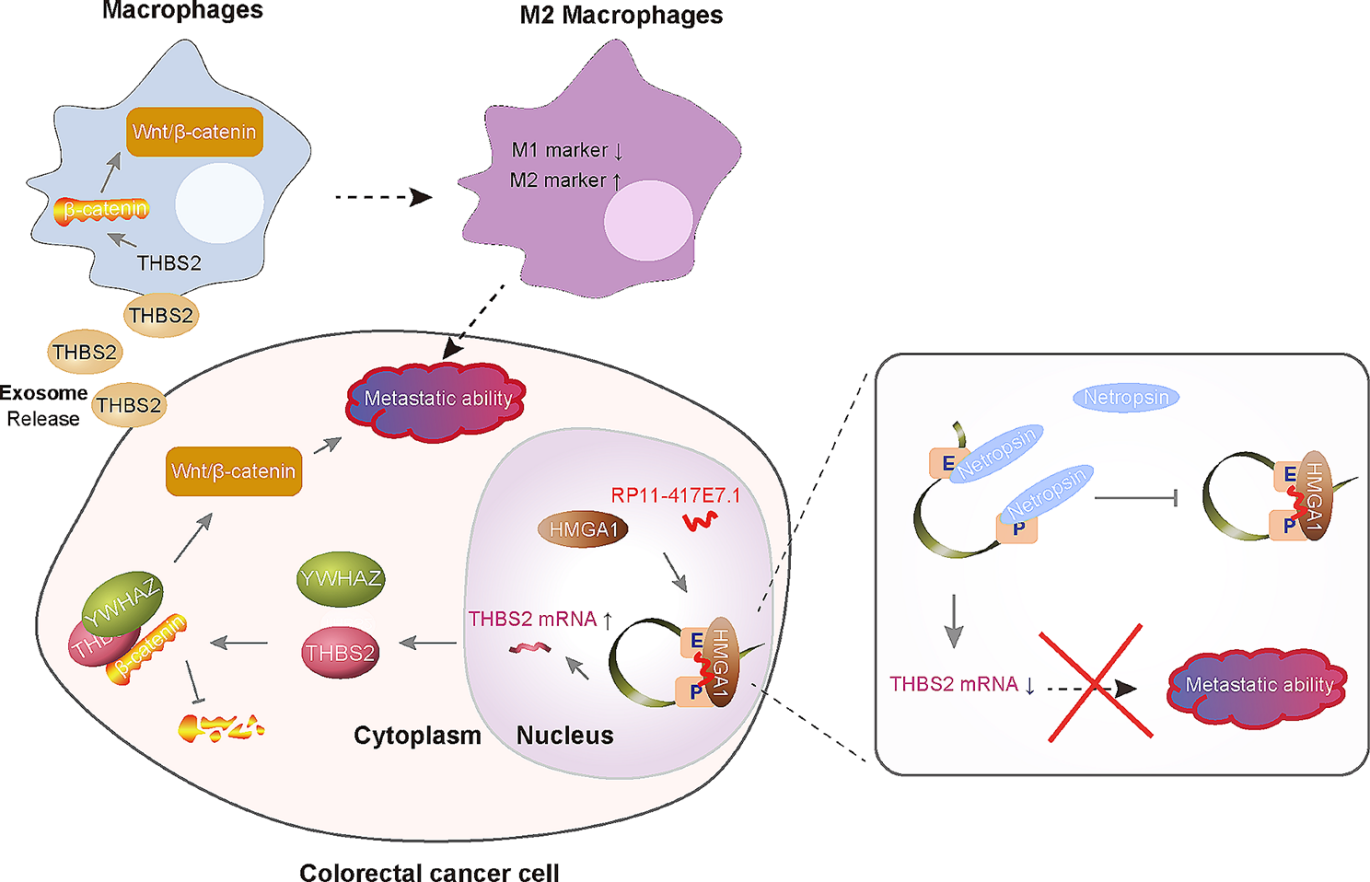
Schematic diagram showing the mechanisms by which RP11-417E7.1 promotes CRC metastasis and netropsin inhibits CRC metastasis. Schematic representation depicting the mechanism by which RP11-417E7.1/THBS2 signaling promotes CRC cell metastasis and the M2 polarization of macrophages. Netropsin inhibits CRC metastasis by blocking the effect of the RP11-417E7.1/HMGA1 complex

We identified the metastasis-associated lncRNA RP11-417E7.1, which has prognostic value. RP11-417E7.1 enhances THBS2 transcription by recruiting HMGA1 to sustain enhancer–promoter looping, thus activating Wnt/β-catenin pathway-mediated metastasis. CRC cells also generate THBS2-rich exosomes that activate Wnt/β-catenin signaling in TAMs, thus inducing the M2 polarization of macrophages to promote the malignant behavior of CRC cells. Furthermore, we found that netropsin can interfere with the role of the RP11-417E7.1-HMGA1 complex in THBS2 transcription, thus disrupting RP11-417E7.1/THBS2 signaling and inhibiting CRC metastasis (Figs. 8).

### Electronic supplementary material

Below is the link to the electronic supplementary material.

Supplementary Material 1

Supplementary Material 2

Supplementary Material 3

## Data Availability

The data generated in this study are available upon request from the corresponding author.

## Abbreviations

CRC: Colorectal Cancer
EMT: Epithelial–Mesenchymal Transition
TAMs: Tumor-Associated Macrophages
lncRNAs: Long Noncoding RNAs
THBS2: Thrombospondin 2
DElncRNA: Differentially Expressed LncRNA
OS: Overall Survival
DFS: Disease-Free Survival
KEGG: Kyoto Encyclopedia of Genes and Genomes
GSEA: Gene Set Enrichment Analysis
qRT–PCR: Real-Time Quantitative Reverse Transcription PCR
ceRNAs: Competing Endogenous RNAs
PRM: Parallel Reaction Monitoring
TFs: Transcription Factors
ChIRP: Chromatin Isolation by RNA Purification
MS: Mass Spectrometry
RIP: RNA Immunoprecipitation
ChIP: Chromatin Immunoprecipitation
3C: Chromosome Conformation Capture
Co-IP: Coimmunoprecipitation
IHC: Immunohistochemistry
