# Supplementary figures and images for: The RP11-417E7.1/THBS2 signaling pathway promotes colorectal cancer metastasis by activating the Wnt/β-catenin pathway and facilitating exosome-mediated M2 macrophage polarization
Source: J Exp Clin Cancer Res. 2024 Jul 17;43:195. doi: 10.1186/s13046-024-03107-7 (PMC11253389; doi:10.1186/s13046-024-03107-7)

proteins: 21  
interactions: 74  
expected interactions: 24 (p-value: 3.33066907387547e-16)

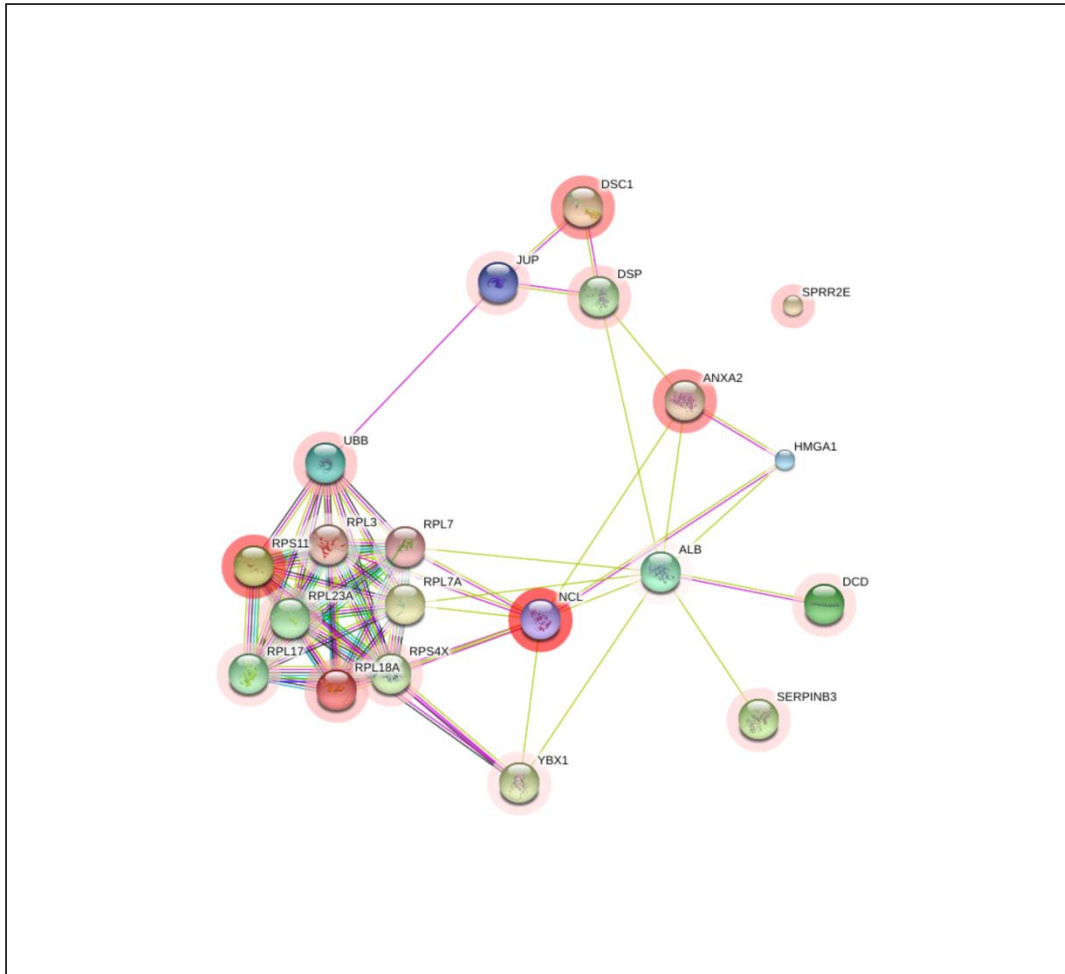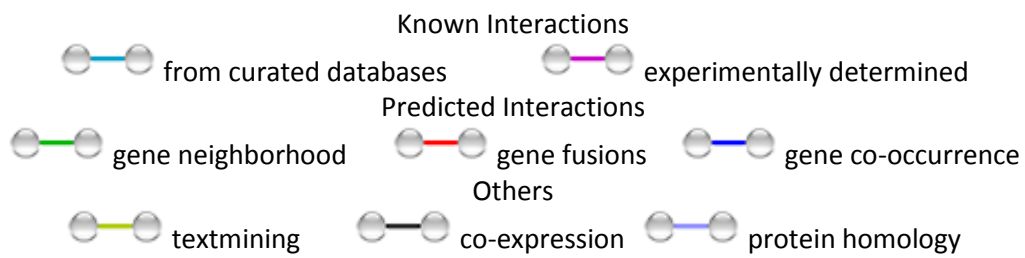

Supplement: Supplementary file 1 — Supplementary Material 1 [file 13046_2024_3107_MOESM1_ESM.pdf]
