# Supplementary material for: The RP11-417E7.1/THBS2 signaling pathway promotes colorectal cancer metastasis by activating the Wnt/β-catenin pathway and facilitating exosome-mediated M2 macrophage polarization
Source: J Exp Clin Cancer Res. 2024 Jul 17;43:195. doi: 10.1186/s13046-024-03107-7 (PMC11253389; doi:10.1186/s13046-024-03107-7)
Supplement: Supplementary file 2 — Supplementary Material 2 [file 13046_2024_3107_MOESM2_ESM.docx]

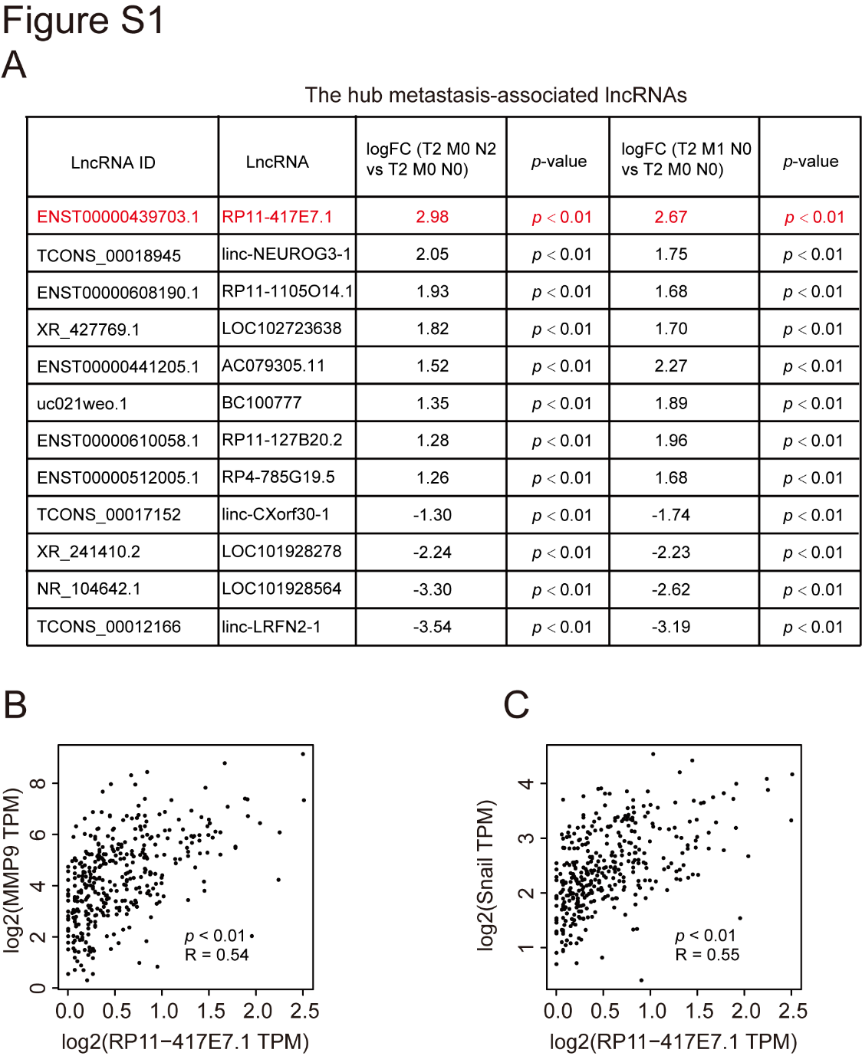


**Figure S1. A,** Schematic representation of the metastasis-asscociated lncRNAs in the collected CRC tissues. **B,** Dot plot of the correlation between RP11-417E7.1 and MMP9 mRNA in the TCGA CRC dataset. **C,** Dot plot of the correlation between RP11-417E7.1 and Snail mRNA in the TCGA CRC dataset.


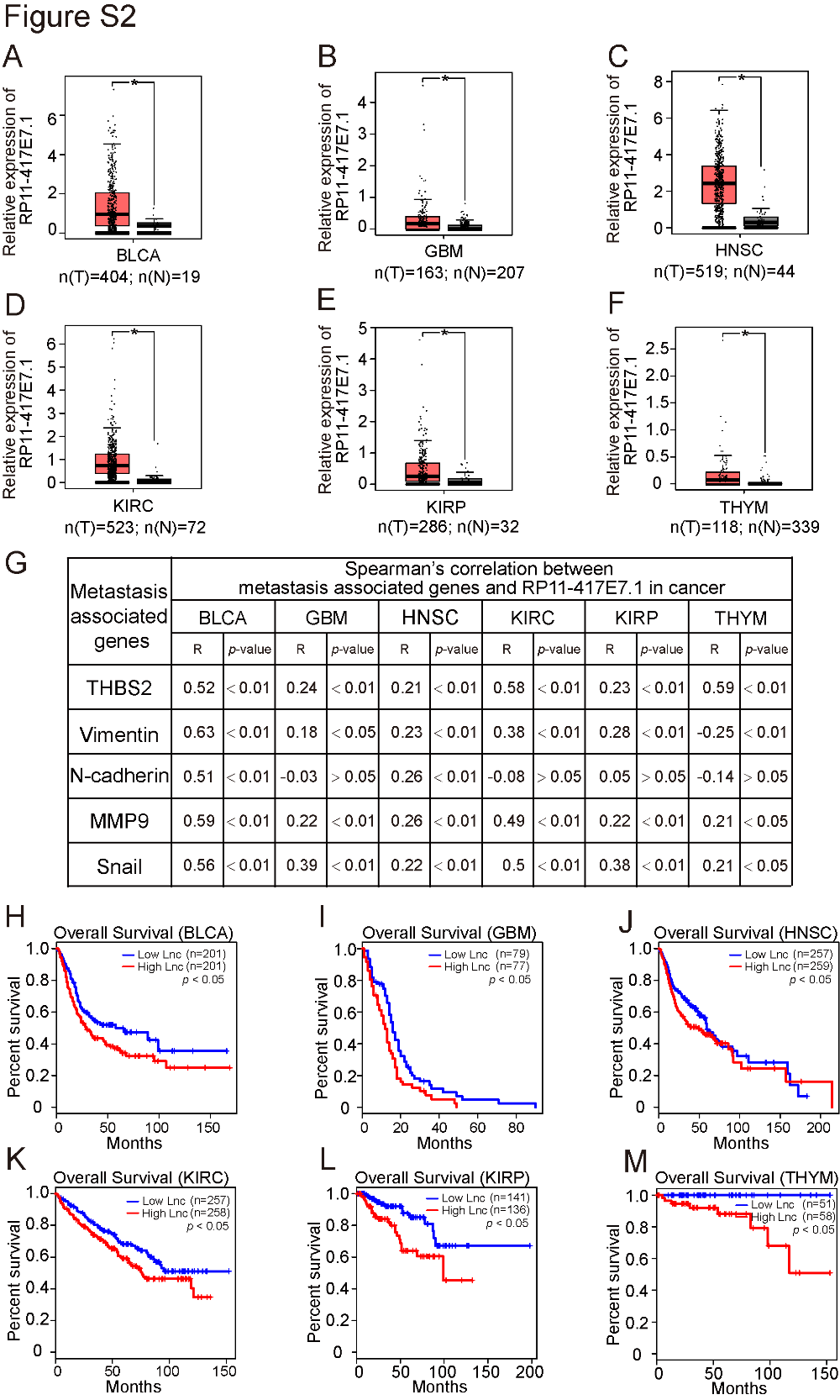


**Figure S2. A-F,** Box plot of RP11-417E7.1 expression across the pan‐cancer tumor types (*, *p* < 0.05). **G,** Correlations between the expression of RP11-417E7.1, THBS2, and metastasis-related genes across multiple tumor types. **H-M,** Kaplan‒Meier analysis of the overall survival rates of pan‐cancer patients with high or low RP11-417E7.1 expression (from GEPIA2).

**
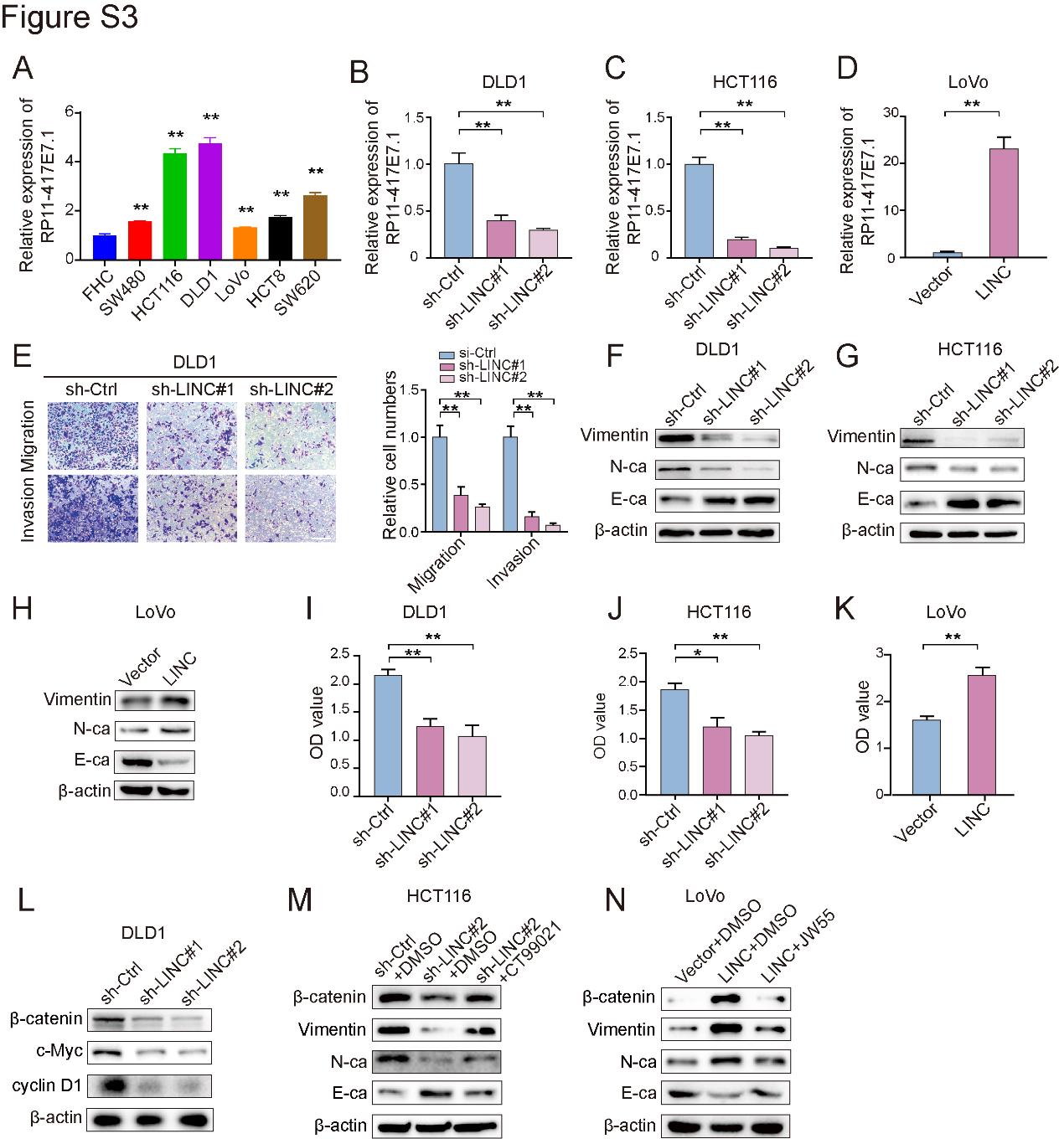
**

**Figure S3. A,** qRT-PCR assays of RP11-417E7.1 in normal and CRC cell lines. **B-D,** qRT-PCR assays of RP11-417E7.1 after knockdown or overexpression. **E,** Transwell assays showed the migratory and invasive potential of RP11-417E7.1-depleted DLD1 cells. Scale bar: 100 μm. **F-H,** Western blot assays of EMT markers in RP11-417E7.1 knockdown or RP11-417E7.1 overexpression cells. **I-K,** Cell adhesion assays showed the adhesion ability of RP11-417E7.1-depleted or RP11-417E7.1-overexpressing CRC cells to the extracellular matrix. **L,** Western blot analysis of β-catenin and downstream targets in RP11-417E7.1-knockdown DLD1 cells. **M,** Western blot analysis of β-catenin and EMT-related proteins from the control, shRP11-417E7.1, and shRP11-417E7.1+CT99021 groups. **N,** Western blot analysis of β-catenin and EMT-related proteins from control, RP11-417E7.1, and RP11-417E7.1+JW55 groups. The data represent the findings from three independent experiments and are shown as the means ± SDs (*, *p* < 0.05; **, *p* < 0.01).


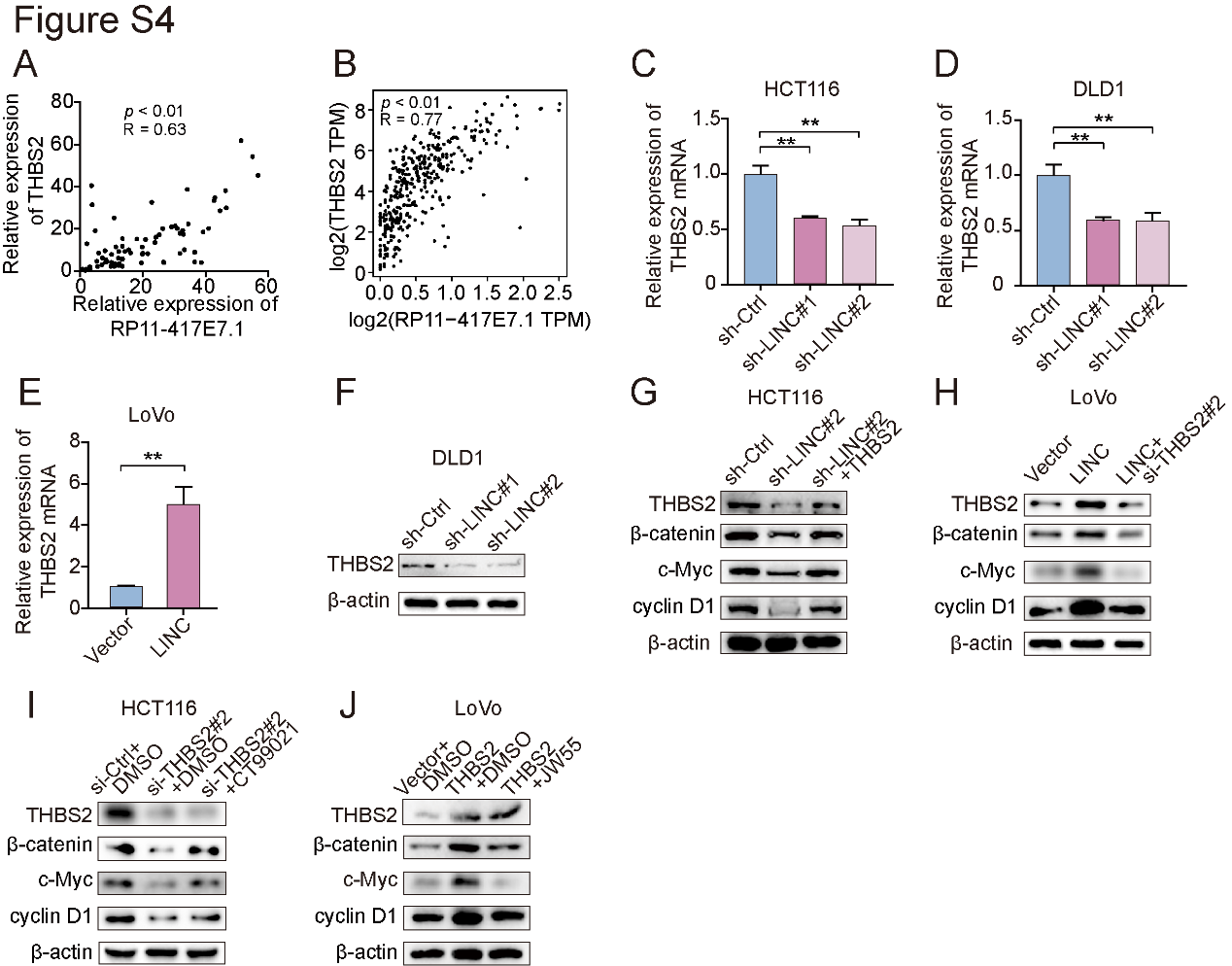


**Figure S4. A,** Dot plot of the correlation between RP11-417E7.1 and THBS2 mRNA expression in the clinical CRC samples. **B,** Dot plot of the correlation between RP11-417E7.1 and THBS2 mRNA in the TCGA CRC dataset. **C-E** qRT-PCR assessment of THBS2 expression in response to RP11-417E7.1 knockdown (C-D) or overexpression (E) in CRC cells. **F,** Western blot analysis of THBS2 after RP11-417E7.1 silencing in DLD1 cells. **G,** Western blot assays of β-catenin and downstream targets in RP11-417E7.1-knockdown CRC cells with THBS2 overexpression or not. **H,** Western blot assays of β-catenin and downstream targets in RP11-417E7.1-overexpressing CRC cells treated with THBS2-siRNA or not. **I,** Western blot assays of β-catenin and downstream targets in si-THBS2 CRC cells treated with CT99021 or DMSO. **J,** Western blot assays showed β-catenin and downstream targets in THBS2-overexpressing CRC cells treated with JW55 or DMSO. The data represent the findings from three independent experiments and are shown as the means ± SDs (*, *p* < 0.05; **, *p* < 0.01).


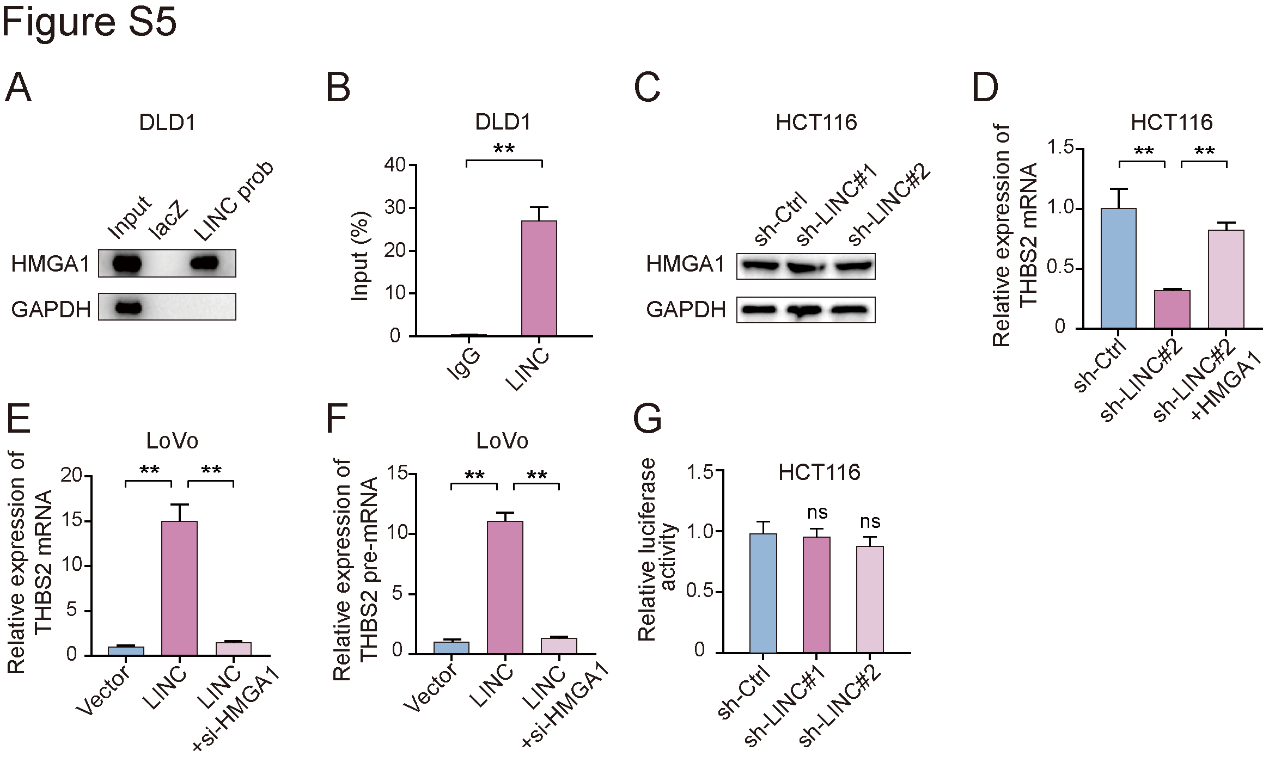


**Figure S5. A,** Western blot assay of HMGA1 protein after ChIRP using RP11-417E7.1 probes in DLD1 cells. **B,** RIP and qRT-PCR analysis showed the enrichment of RP11-417E7.1 after the immunoprecipitation of HMGA1 in DLD1 cells. **C,** Western blot assay of HMGA1 protein after RP11-417E7.1 knockdown in HCT116 cells. **D,** qRT-PCR analysis showed THBS2 mRNA expression in RP11-417E7.1-depleted HCT116 cells treated with HMGA1 plasmid or vector control. **E-F,** qRT-PCR assays showed THBS2 mRNA and pre-mRNA levels in RP11-417E7.1-overexpressing CRC cells treated with siRNA ctrl or siHMGA1. **G,** The promoter activity of THBS2 after RP11-417E7.1 knockdown in HCT116 cells. The data represent the findings from three independent experiments and are shown as the means ± SDs (*, *p* < 0.05; **, *p* < 0.01).


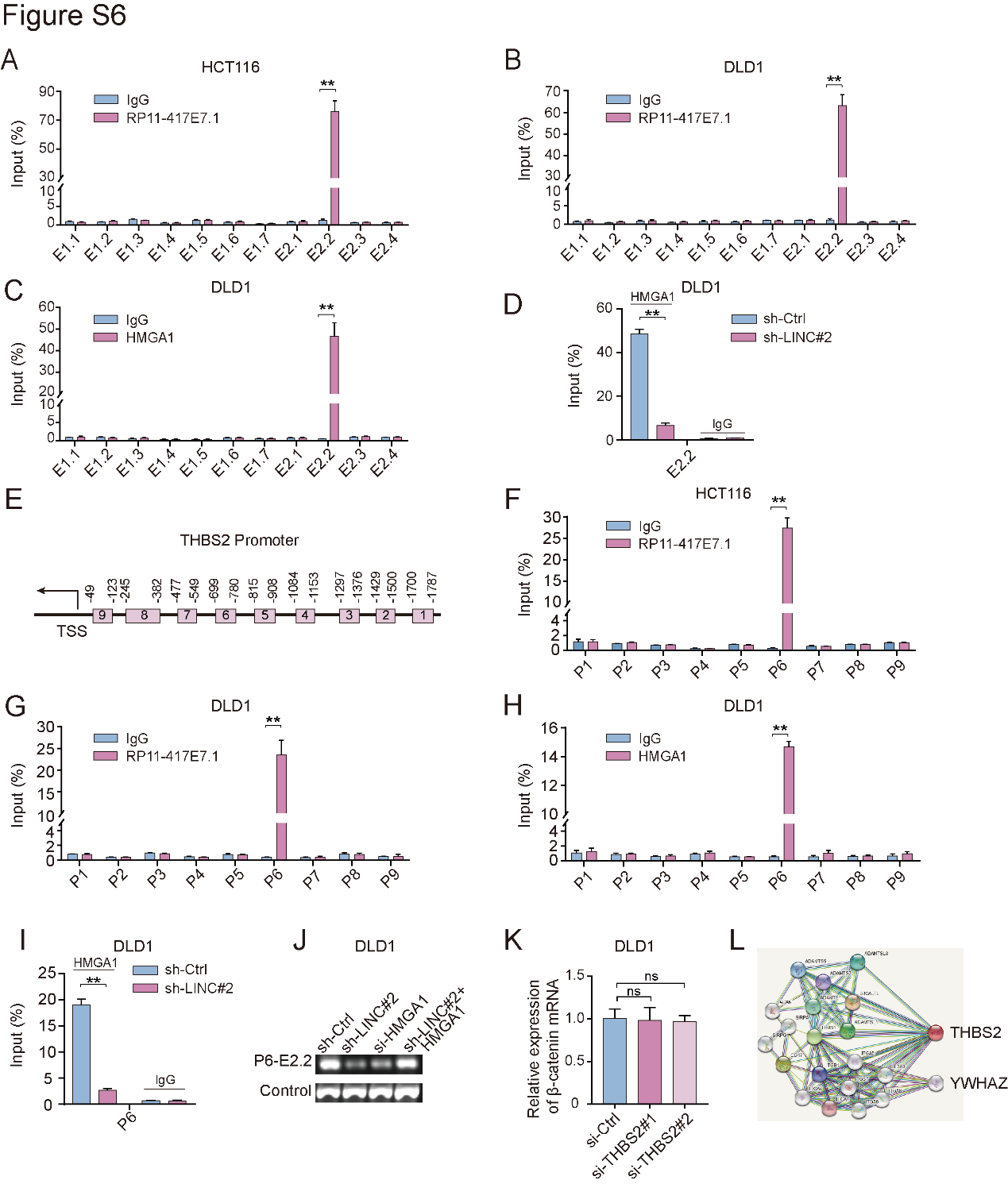


**Figure S6 A-B,** ChIRP-qPCR assay showing the enrichment of RP11-417E7.1 at the enhancer areas of THBS2 when different primers were used in CRC cells. **C,** ChIP-qPCR results showed the enrichment of HMGA1 at the enhancer areas of THBS2 in DLD1 cells. **D,** ChIP-qPCR showed changes in HMGA1 recruitment at the E2.2 area after RP11-417E7.1 knockdown in DLD1 cells. **E,** A schematic of the designed primers targeting THBS2 promoter regions. Red boxes indicate the ChIP-qPCR primers targeting the promoter regions. TSS indicates the transcription start site. **F-G,** ChIRP-qPCR assays showed the enrichment of RP11-417E7.1 at the promoter of THBS2 in CRC cells; primers of the promoter region (P1-P9) were used. **H,** ChIP-qPCR assays showed the enrichment of HMGA1 at the promoter of THBS2 in DLD1 cells. **I,** ChIP-qPCR revealed changes in HMGA1 recruitment at the promoter of THBS2 after silencing of RP11-417E7.1 in DLD1 cells. **J,** 3C assays of the chromatin loop structure between THBS2 enhancer E2.2 and promoter P6 locus in DLD1 cells from the control, shRP11-417E7.1, siHMGA1, and RP11-417E7.1+siHMGA1 groups. **K,** qRT-PCR assay of β-catenin mRNA expression in si-THBS2-transfected DLD1 cells. **L,** Schematic diagram of protein-protein interactions between THBS2 and YWHAZ (STRING database). The data represent the findings from three independent experiments and are shown as the means ± SDs (*, *p* < 0.05; **, *p* < 0.01).

**
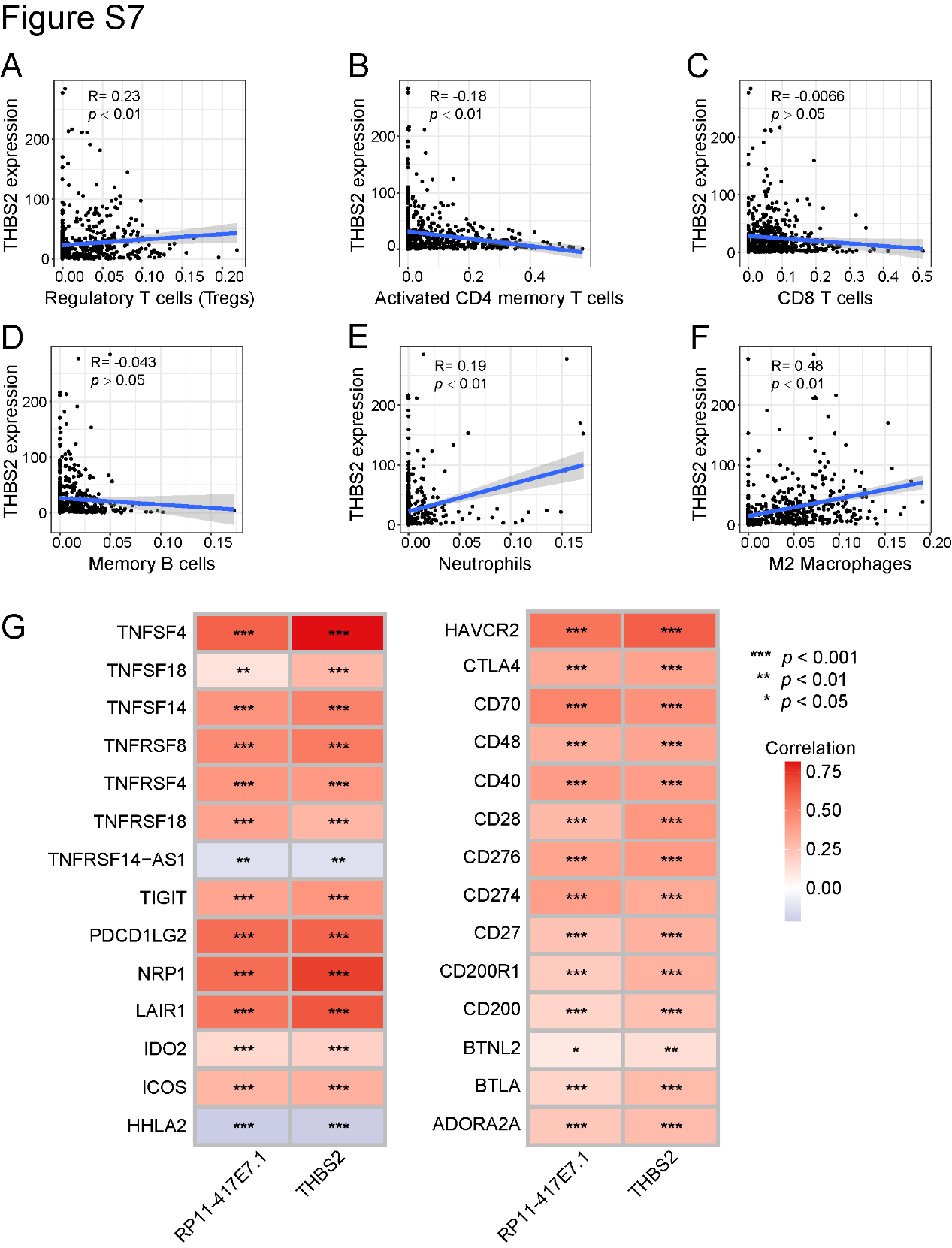
**

**Figure S7.** Dot plot of the correlation between mRNA expression level of THBS2 and infiltrating immune cells that calculated based on the CIBERSORT method in the CRC samples from TCGA, including regulatory T cells (Tregs) (**A**), activated CD4+ memory T cells (**B**), CD8+ T cells (**C**), memory B cells (**D**), neutrophils (**E**) and M2 macrophages (**F**). **G,** Heatmap of correlation between RP11-417E7.1/THBS2 expression and immune checkpoint genes expression using the CRC RNA-Seq data from TCGA. *, *p* < 0.05; **, *p* < 0.01; ***, *p* < 0.001.


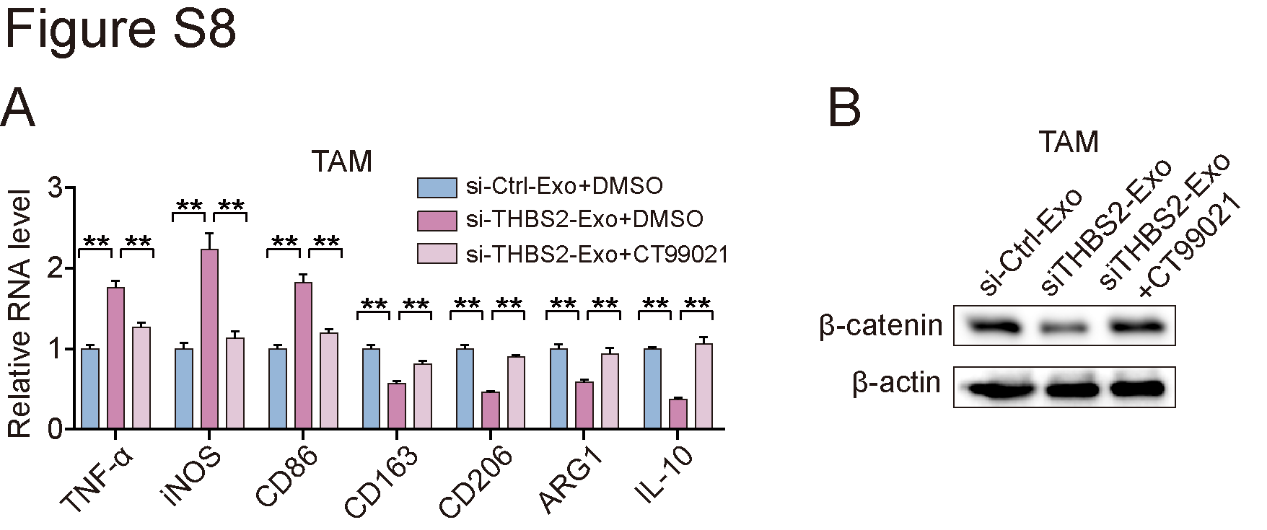


**Figure S8. A-B,** Macrophages were added with exosomes (10 μg) isolated from supernatants of si-Ctrl or si-THBS2-transfected HCT116 cells. 24h after exosome treatment, macrophages were treated with or without CT99021. Then, M1 and M2 markers were detected by qRT-PCR analysis (A), and β-catenin was detected by western blot analysis (B). The data represent the findings from three independent experiments and are shown as the means ± SDs (*, *p* < 0.05; **, *p* < 0.01).
