# Supplementary material for: The RP11-417E7.1/THBS2 signaling pathway promotes colorectal cancer metastasis by activating the Wnt/β-catenin pathway and facilitating exosome-mediated M2 macrophage polarization
Source: J Exp Clin Cancer Res. 2024 Jul 17;43:195. doi: 10.1186/s13046-024-03107-7 (PMC11253389; doi:10.1186/s13046-024-03107-7)
Supplement: Supplementary file 3 — Supplementary Material 3 [file 13046_2024_3107_MOESM3_ESM.docx]

**Supplemental Materials and Methods**

**Microarray and RNA-seq assay**

Cancer cells was extracted and maintained in the TRIzol (Invitrogen, Shanghai, China). Microarray experiments were performed by KangChen (Shanghai, China). An Arraystar Human lncRNA Microarray V3.0 (Rockville, MD, USA) was used. The detailed steps of microarray such as RNA extraction, cDNA synthesis, and labeling reaction were performed according to the protocol. Preparation and transcriptome sequencing were conducted at Huada (Beijing, China) through an MGISEQ-2000 platform, generating 100-bp paired-end reads. The number of reads mapped per gene was calculated using MGISEQ-2000 for the number of mapped fragments per million transcribed kilobases (FPKM) per gene. Differences in gene expression were expressed as FC (fold changes).

**RNA extraction and Quantitative real-time PCR (****qRT-PCR) assay**

Total RNA of cells or tissues were extracted RNA isolater Total RNA Extraction Reagent (Vazyme, Nanjing, China) following the manufacturer’s instructions. cDNA was prepared for qPCR using HiScript Q RT SuperMix (Vazyme, Nanjing, China), and qPCR was conducted using UltraSYBR Mixture (Cwbio, Beijing, China). 2-ΔΔCt was used to quantify gene expression, and GAPDH was used for normalization.

**RNA-fluorescence in situ hybridization (****RNA FISH)**

Cells were washed twice with PBS and then fixed in 4% formaldehyde for 15 min at room temperature. The fixed cells were permeabilized with 0.5% Triton X-100/PBS for 5 min at 4 °C and then prehybridized and hybridized with fluorescent probes at 37°C overnight. On the next day, cells were stained with DAPI and observed under a confocal microscope (Olympus LSM488, Tokyo, Japan). Specific probes targeting lncRNA RP11-417E7.1 were subscribed from (GenePharma, Shanghai, China). FISH kit was purchased from RiboBio Co., Ltd. (Guangzhou, China).

**Immunofluorescence (****IF) analysis**

Cells were fixed with 4% paraformaldehyde for 10 min and washed with PBS three times. Then, cells were permeabilized using 0.5% Triton X-100/PBS. Next, the cells were blocked using 5% BSA (Solarbio, Beijing, China) incubated with specific primary antibody and secondary antibody, followed by staining with DAPI. Samples were kept in dark place and images were captured on a confocal microscope.

**RNA immunoprecipitation (****RIP) assay**

RIP experiments were carried out using a RIP Kit (Merck Millipore, Burlington, MA, USA) according to the protocol. Briefly, the specific antibody or anti-IgG (negative control) was prebound to magnetic beads. Cell lysates were then incubated with beads. After elution from magnetic beads, RNA products were obtained and detected by qPCR.

**Chromatin isolation by RNA purification (ChIRP)**

ChIRP assay was performed using a ChIRP Kit (Bersinbio, Guangzhou, China) under the manufacturer’s guidelines. Briefly, cells were cross-linked with paraformaldehyde and then lysed by sonication. Next, the cell lysates were hybridized in hybridization buffer with biotin-labelled probes. Finally, bound DNA products or proteins were extracted. ChIRP-mass spectrometry (ChIRP-MS) was performed by KangChen (Shanghai, China). The bound DNA products were further confirmed by RT-qPCR. Captured proteins were analyzed by Western blot.

**Chromatin immunoprecipitation (ChIP)**

A ChIP assay was performed using a ChIP assay kit (Millipore, Billerica, MA, USA, USA) according to the manufacturer’s instructions. In brief, cells were cross-linked with paraformaldehyde and then lysed by sonication to shear and obtain DNA fragments. Next, the samples were subjected to IP using specific antibodies or negative control (anti-IgG). After washing with a series of buffers, purified DNA fragments were detected by qPCR. The primers of DNA loci are listed in Supplemental Materials and Methods.

**Chromatin conformation capture (3C) assays**

3C assays were performed as previously described (1). Briefly, cells were trypsinized and then crosslinked with 1% formaldehyde, followed by quenching it with 0.125 M glycine on ice for 10 min. After cell lysis, the nuclei were harvested and suspended in digestion buffer (0.3% SDS and 2% Triton X-100). The chromatin was digested with Csp6I at 37°C. Then, chromatin ligation was incubated with T4 ligase (New England Biolabs, Hitchin, UK) for 4 h at 16°C, followed by incubation with Proteinase K (Sigma, USA) overnight at 65°C to reverse the cross-links. The samples were treated with RNase A, and DNA ligation products were purified by phenol/chloroform extraction. The purified DNA ligation products were detected by PCR analysis. The primer was listed in Supplemental Materials and Methods.

**Dual luciferase reporter assay**

The regions of THBS2 promoter and lncRNA RP11-417E7.1 promoter were PCR amplified and cloned into the pGL3 vector (Promega, Madison, WI, USA). Cells were cotransfected with the constructed luciferase reporter, a renilla luciferase control plasmid and siRNA (or overexpression vector). After 48 h incubation, the luciferase activity was measured with a Dual-Luciferase Reporter Assay System (Promega, Madison, WI, USA).

**TOPFlash reporter assay**

The TOPFlash and FOPFlash (negative control) luciferase reporters were purchased from Addgene (Cambridge, MA, USA). Briefly, cells were co-transfected with TOPFlash or FOPFlash firefly luciferase reporter, and Renilla luciferase control reporter. After 24 h of transfection, cells were lysed and luciferase activity was measured using the Dual-Luciferase Reporter Assay Kit (Promega, Madison, WI, USA). The TOPFlash or FOPFlash activity was normalized to Renilla luciferase signals.

**Western blot analysis and antibodies**

Cells were lysed in RIPA buffer containing proteinase inhibitors. Protein concentration was detected using BCA Protein Assay Kit (Beyotime, Shanghai, China). Equal amounts of protein were subjected to Western blot analysis. Total proteins were separated on a 4–12% sulfate-polyacrylamide gel and then transferred onto a nitrocellulose membrane. Membranes were incubated at 4℃ overnight with primary antibodies in 3% BSA in TBST, followed by incubation with secondary antibodies for 1 hour at room temperature. Western blot signals were visualized by ECL plus Western blotting detection system (Vazyme, Nanjing, China). Antibodies used are as follows: anti-GAPDH (Abcam, ab8245), anti-THBS2 antibody (Abcam, ab84469), anti-c-Myc antibody (Abcam, ab32072), anti-E-Cadherin antibody (Proteintech, 20874-1-AP), Anti-Vimentin antibody (Proteintech, 10366-1-AP), anti- Cyclin D1 antibody (Proteintech, 26939-1-AP), anti- β-actin antibody (Proteintech, 20536-1-AP), anti-β-catenin antibody (Abcam, ab32572), Anti-N Cadherin antibody (Abcam, ab76011), anti-HMGA1 antibody (Abcam, ab252930), anti-YWHAZ antibody (Abcam, ab155037), anti- Histone H3 antibody (Abcam, ab1791), anti-Flag antibody (Proteintech, 20543-1-AP), anti-HA antibody (51064-2-AP), anti-His antibody (Proteintech, 66005-1-Ig), anti-CD163 antibody (Proteintech, 16646-1-AP), and anti-β-TrCP antibody (ab71753).

**Co-inmunoprecipitation (Co-IP)**

Collected cells were lysed with IP Lysis Buffer (Beyotime, Shanghai, China). After centrifugation, the cell lysate supernatant was collected and incubated with primary antibodies at 4℃ overnight. On day 2, the mixture was precipitated with magnetic beads for 2 hours. Then, IP proteins were eluted from the magnetic beads using 2 × Loading Buffer (Beyotime, Shanghai, China), and visualized by Western blot.

**Cell migration and invasion assay**

Cells were harvested and re-suspended in serum-free medium. For the migration assay, the above cells were placed in the upper chambers of transwell plates (Corning, New York, NY, USA). For the invasion assay, the cells were plated to the transwell upper chambers precoated with Matrigel. The chambers were then placed into 24-well plates containing cell growth medium (20% FBS). After 24 h (migration assay) or 48 h incubation (invasion assay), cells were fixed with 4% paraformaldehyde and immersed into 0.1% crystal violet solution. Stained cells were then visualized under microscope.

**Cell adhesion assays**

Cell adhesion assays were performed using the CytoSelect 48-well Cell Adhesion Assay Kit (Cell Biolabs, San Diego, CA) according to the protocol. In brief, each well was inoculated with 1.5×10^5^ cells and incubated in a cell culture incubator for 1 h. Next, it needs to carefully remove the medium (containing the nonadherent cells) and clean the wells, followed by staining and lysing. Finally, the absorbance was measured using a plate reader (OD = 560 nm).

**Macrophage polarization experiments**

THP-1 cells were treated with PMA (Sigma-Aldrich, Saint Louis, MO, USA) for 24 h to induce macrophage-like differentiation. To mimic TAMs formation, macrophages were co-cultured with CRC cells in a 6-well transwell co-cultivation system (Corning, New York, NY, USA). CRC cells were added to the upper chamber, and the macrophages were added to the lower chamber. After incubation for 48 h, the co-cultured macrophages were collected as TAMs.

**Exosome isolation and purification**

Exosome isolation from cell medium (CM) was performed by differential ultracentrifugation. In brief, CM was collected and centrifuged at 300 × g for 10 min, followed by 2000 × g for 10 min and 10 000 × g for 30 min. Then, exosomes were obtained from CM by 100 000 × g ultracentrifugation for 70 min. The pellets were washed with PBS, and ultracentrifuged at 100,000 × g for another 70 min. All centrifugation steps were performed at 4 °C. The morphology of the collected exosomes was observed using transmission electron microscopy (TEM).

**Exosome labelling and fluorescence microscopy**

The CRC cell-derived exosomes were incubated with the green fluorescent dye DiO at 37 °C for 20 min, and the labelled exosomes were centrifugated and washed three times. Then, the DiO-labeled exosomes were incubated with macrophages, followed by fixation with 4% paraformaldehyde. Finally, the fluorescence signals were acquired using confocal microscope (Zeiss LSM800, Oberkochen, Germany).

**Immunohistochemistry (IHC)**

IHC was performed according to the manufacturer’s protocol of the SP Kit (ZSGB-bio, Beijing, China). In brief, tissues were fixed with 4% formalin, embedded in paraffin, and cut into 5-µm sections. Sections were deparaffinized in a series of xylene and gradient alcohol, followed by antigen retrieval using boiling citrate buffer. Then, the sections were blocked with blocking buffer, incubated using primary antibody at 4°C overnight. On day 2, samples were incubated with HRP-conjugated secondary antibody at room temperature. Finally, each protein was detected using a DAB Kit (ZSGB-bio, China). Images were taken by upright metallurgical microscope (Olympus, Tokyo, Japan).

***In vivo* metastasis models**

All the animal studies were allowed by the Committee on the Ethics of Animal Experiments of Xuzhou Medical University, and complied with the National Institutes of Health Guide for the Care and Use of Laboratory Animals. The maximal tumor diameter permitted by the Animal Care Committee of Xuzhou Medical University is 15 mm and was not exceeded in the experiments.

BALB/c nude mice (six weeks old) were obtained (Shanghai Slac Laboratory Animal Co. Ltd., Shanghai, China), and bred and maintained under specific pathogen free conditions. The mice in the experimental groups or control groups are divided randomly. For the orthotopic CRC model, 1×10^5^ HCT116 cells were injected into the mice cecum after laparotomy. The mice were randomized into two groups (control group and treatment group). Two weeks later, control mice were treated with vehicle and mice in treatment group were injected with netropsin (Sigma-Aldrich, Saint Louis, MO, USA; 0.2 mg/kg every two days) by intraperitoneal route, respectively. Six weeks after the injection of CRC cells, metastases were evaluated by *In Vivo Imaging System* (IVIS), and mice were sacrificed.

For the lung metastasis assay, 1×10^6^ HCT116 cells suspended in 200 μl PBS were injected into the tail vein of mice (In netropsin treatment experiments, mice were treated with physiological saline or 0.2 mg/kg netropsin intraperitoneally every two days after tumor injection). All the mice were sacrificed for analysis of lung metastasis after five weeks since injection. The number of metastatic nodules were calculated and the lungs were then fixed in 4% paraformaldehyde for immunohistochemistry.

For intraspleen injection, 1×10^6^ HCT116 cells suspended in 50 μl PBS were injected into the mice spleen after laparotomy, and the spleen was removed after 10 min. Six weeks after the injection of CRC cells, metastases were evaluated by *In Vivo Imaging System* (IVIS), and mice were sacrificed.

1. Hagège H, Klous P, Braem C, Splinter E, Dekker J, Cathala G, et al. Quantitative analysis of chromosome conformation capture assays (3C-qPCR). Nat Protoc. 2007;2(7):1722-33.

**Primers for qRT-PCR**

| Transcript | Forward | Reverse |
| --- | --- | --- |
| THBS2 | ATAGACAGCTTCGCTCTGGAC | CAAACCCCTGAAGTGACTCTC |
| lncRNA RP11-417E7.1 | CTGGCTCCGTCAGAGGATGGG | ACGCAGGCTTGTTGTTGGATGG |
| TNF-α | CAGACTTC CTTGAGACACGG | CAAGGCAGCTCCTACATTGG |
| iNOS | TGCGTTACTCCACCAACAAT | ATGAGCTGAGCATTCCACAC |
| CD86 | CTGCTCATCTATACACGGTTACC | GGAAACGTCGTACAGTTCTGTG |
| CD163 | GCGGGAGAGTGGAAGTGAAAG | GTTACAAATCACAGAGACCGCT |
| IL-10 | GACTTTAAGGGTTACCTGGGTTG | TCACATGCGCCTTGATGTCTG |
| CD206 | CTACAAGGGATCGGGTTTATGGA | TTGGCATTGCCTAGTAGCGTA |
| GAPDH | GACAGTCAGCCGCATCTTCT | TTAAAAGCAGCCCTGGTGAG |
|  |  |  |

**Primers of THBS2 promoter areas:**

|  | Forward | Reverse |
| --- | --- | --- |
| P1 | GAGCTGCCTGTCTTCAGCTT | GCATCCCCTACTCTGCGTG |
| P2 | ACGTACCTTAAACATGACACATACA | GGCAAACATCAGTTTCCGACA |
| P3 | AAGCTCCTTCCCTCTCCCAC | ACCATGAAGAGCAAGCCTCA |
| P4 | CCAGGCTGACAACTCCACAC | ACCTGCACCTGCTTCATGTC |
| P5 | AAACAGCCTCTCCACCATCC | GGGCTGCTTCTCCCAAGTT |
| P6 | GCAGCTTCCCCTTTCCACT | CTTCTCAGAGGTCAGGGTGG |
| P7 | ACGTGCAAGTGTGCACCAAG | AATACCCGTAGAAGGGTGTGTC |
| P8 | GCTTTTCAGTCCTCTAACTGCC | CGGTGAGTATCAATAATCAGTCTGT |
| P9 | AGTTCTAGCCAATCTTGCTAGTGT | GCAGGGACTCACTTTTCCTGT |
|  |  |  |

**Primers of THBS2 enhancer areas:**

|  | Forward | Reverse |
| --- | --- | --- |
| E1.1 | AGATGCGGAGAGGCTTTTAGG | CCATGGGCCTCCCTGTAGTAT |
| E1.2 | CCAGCAATGAGTTTTCCCATGA | TGTTAATGATCTACTGTACAGGGC |
| E1.3 | GAGATGGGCTGTGGGATACG | CAGGGTCTCGGCAATGACAG |
| E1.4 | TGTTTTGCAATGACGGAGGC | GTCAACTCCCACACCACGAG |
| E1.5 | TTTGGAAACTGCTGTGGTTGT | CCCATGGCCTGCAATGTTTG |
| E1.6 | TGGGCCCTGAGACAAGAGA | AGGAAGAGACTCTGGACTGCT |
| E1.7 | AGAACAACGCAGGTTAATGTGTAAA | AGTGAGATGTGGGCTGGTAAA |
| E2.1 | TATCATTCTTCCACAAAGGCCAAT | CTGGATGTAAAAGAGTCCCTCCT |
| E2.2 | TGAGATTTGCCCTTCCACCT | CTCCAGCAGTCAGTTGGGTG |
| E2.3 | ATTGTCTCCAGGCCCCAGA | TCACTTCTCCGTCTCCCCAG |
| E2.4 | GGGAGACGGAGAAGTGAGGA | GGTTTCCGCACAGGAATGC |

**Primers of P-E product:**

|  | Forward | Reverse |
| --- | --- | --- |
|  | GTCAGGTTGTTCCTGTGATGG | CAGGCACTATCTTGTGGGTAAAG |

**shRNA and siRNA sequence**

**shlncRNA RP11-417E7.1#1**

CACCGGCTGACATTGTACGAGCCATTCGAAAAAATTGATCAATGCCGAGGA

**shlncRNA RP11-417E7.1#2**

CACCGTCCTCGGCATTGATCAATTTTCGAAAAAATTGATCAATGCCGAGGA

**sh-Ctrl**

CACCGTTCTCCGAACGTGTCACGTTTCGAAAAACGTGACACGTTCGGAGAA

**siTHBS2#1:**

GCAAGAUCACCAAGAUCAUTT

**siTHBS2#2:**

GCGACCUCAUAGACAGCUUTT

**si-YWHAZ:**

GCCUGCAUGAAGUCUGUAATT

**si-Ctrl**

UUCUCCGAACGUGUCACGUTT

**si-HMGA1**

CCACCUGCUCCUUAGAGAAGG
